# Dynamic roles of small RNAs and DNA methylation associated with heterosis in allotetraploid cotton (*Gossypium hirsutum* L.)

**DOI:** 10.1186/s12870-023-04495-2

**Published:** 2023-10-13

**Authors:** Rasmieh Hamid, Feba Jacob, Zahra Ghorbanzadeh, Leila Jafari, Omran Alishah

**Affiliations:** 1 Department of Plant Breeding, Cotton Research Institute of Iran (CRII), Agricultural Research, Education and Extension Organization (AREEO), Gorgan, Iran; 2 Centre for Plant Biotechnology and Molecular Biology, Kerala Agricultural University, Thrissur, India; 3 Department of Systems Biology, Agricultural Biotechnology Research Institute of Iran (ABRII), Agricultural Research, Education and Extension Organization (AREEO), Karaj, Iran; 4 Horticultural Science Department, Faculty of Agriculture and Natural Resources, University of Hormozgan, Bandar Abbas, Iran; 5 Research Group of Agroecology in Dryland Areas, University of Hormozgan, Bandar Abbas, Iran

**Keywords:** Cotton, Methylome, Small RNA

## Abstract

**Background:**

Heterosis is a complex phenomenon wherein the hybrids outperform their parents. Understanding the underlying molecular mechanism by which hybridization leads to higher yields in allopolyploid cotton is critical for effective breeding programs. Here, we integrated DNA methylation, transcriptomes, and small RNA profiles to comprehend the genetic and molecular basis of heterosis in allopolyploid cotton at three developmental stages.

**Results:**

Transcriptome analysis revealed that numerous DEGs responsive to phytohormones (auxin and salicylic acid) were drastically altered in F1 hybrid compared to the parental lines. DEGs involved in energy metabolism and plant growth were upregulated, whereas DEGs related to basal defense were downregulated. Differences in homoeologous gene expression in F1 hybrid were greatly reduced after hybridization, suggesting that higher levels of parental expression have a vital role in heterosis. Small RNAome and methylome studies showed that the degree of DNA methylation in hybrid is higher when compared to the parents. A substantial number of allele-specific expression genes were found to be strongly regulated by CG allele-specific methylation levels. The hybrid exhibited higher 24-nt-small RNA (siRNA) expression levels than the parents. The regions in the genome with increased levels of 24-nt-siRNA were chiefly related to genes and their flanking regulatory regions, demonstrating a possible effect of these molecules on gene expression. The transposable elements correlated with siRNA clusters in the F1 hybrid had higher methylation levels but lower expression levels, which suggest that these non-additively expressed siRNA clusters, reduced the activity of transposable elements through DNA methylation in the hybrid.

**Conclusions:**

These multi-omics data provide insights into how changes in epigenetic mechanisms and gene expression patterns can lead to heterosis in allopolyploid cotton. This makes heterosis a viable tool in cotton breeding.

**Supplementary Information:**

The online version contains supplementary material available at 10.1186/s12870-023-04495-2.

## Background

Upland cotton (*Gossypium hirsutum*) is a premium fiber, a source of edible oil, and one of the world’s most economic crops [1]. The demand for cotton fiber in the textile industry is increasing, but the supply is decreasing due to the small area under cultivation and the different yield potential of the varieties [2]. Plant breeders are working hard to overcome these obstacles to increase yields and resilience to biotic and abiotic stresses. In this context, heterosis breeding has led to significant progress [3].

The superior performance of F1 hybrid plants over their parents is known as heterosis or hybrid vigour [4]. Heterosis is widely used in commercial plant breeding to achieve better traits such as higher developmental rate, biomass production, grain yield, and stress tolerance [5, 6]. Despite increasing research data on cotton heterosis, the basic molecular processes that trigger heterosis are still largely not understood [6, 7]. Traditional genetic explanations include the ideas of epistasis, dominance, and overdominance [8]. However, the molecular basis of heterosis cannot be elucidated by these hypotheses and they do not suit the genome-level data. Several studies have shown that genomic and epigenetic alterations are involved in hybrid heterosis [9]. The modifications in histones and DNA methylation are epigenetic processes that affect gene expression while there is no change in the DNA sequence [10]. DNA methylation is a well-studied epigenetic process in plants, which occurs when a methyl group is attached to the 5’-carbon of the pyrimidine ring of cytosine nucleotides [11]. In plants, cytosine methylations are divided into CHH, CHG and CG contexts (H can be any nucleotide other than G) [12].

In addition to DNA methylation and histone modification, small RNAs like microRNAs (miRNAs), trans-acting ta-siRNAs, and small interfering RNAs (siRNAs) also have vital roles in epigenetics [13]. The initiation and maintenance of DNA methylation in plants are mediated by RNA-directed DNA methylation (RdDM), in all sequence contexts [14]. Small interfering RNAs (siRNAs) drive Domains Rearranged Methyltransferase 2 (DRM2), the de novo DNA methyltransferase, to methylation sites in genomic DNA. siRNAs affect gene expression by modulating DNA methylation, which is particularly important in transposon elements (TEs) and some protein-coding genes. Trans- and cis-regulatory miRNAs, on the other hand, regulate spontaneous variations in a variety of metabolic pathways that affect genome-wide methylation patterns [15, 16]. Numerous studies have been showed to examine the global patterns of naturally occurring variation in small RNAs and epigenetic modifications, as well as the resulting effect on diversification of the transcriptome [17, 18]. For instance a group of researchers used Arabidopsis epiRILs with different degrees and distributions of DNA methylation, to demonstrate their impact on the genetic regulation of heterosis [19]. These epigenetic modifications in hybrids influence phenotypic aspects as well as biological pathways such as metabolism, growth, biomass, as well as hormone signaling, response to stress and senescence, flowering, fruiting, and yield [20].

The goal of this study was to conduct a detailed investigation of elite cotton hybrid and its parental lines through transcriptome sequencing, bisulfite sequencing, small RNA profiling and genome resequencing. The methylation patterns of CG, CHG, and CHH in cotton seedlings were examined and differentially methylated regions (DMRs) were compared between the hybrid and its parents. The patterns of methylation and the density of small RNA in the gene body and surrounding 5’ and 3’ regions of protein coding and TEs were compared with the expression level of TE and the genes. The results suggest that transcriptional and epigenetic changes, as well as genome-wide reorganization of gene expression in the hybrid, play a key role in hybrid vigour.

## Results

### F1 hybrid shows significant morphological heterosis

The F1 hybrid, resulting from crossing Latif (maternal line) with Taban (paternal line), has a significantly higher number of bolls and higher fibre yield (Table S1). The total seed cotton yield of the F1 line was 16% higher than the mean of the parents (MPV). The higher number of bolls (about 20% higher than MPV) was the direct result of a significant increase in fruiting branches between F1 and the two parental lines, but it wasn’t due to a longer flowering period because the flowering period of F1 was between the two parental lines and all three genotypes were harvested simultaneously. Based on the increase in yield heterosis and its stability during two years of field experiments, this hybrid can be considered suitable for studying the comparative genomics and the mechanisms controlling yield heterosis in cotton.

**Fig. 1:**
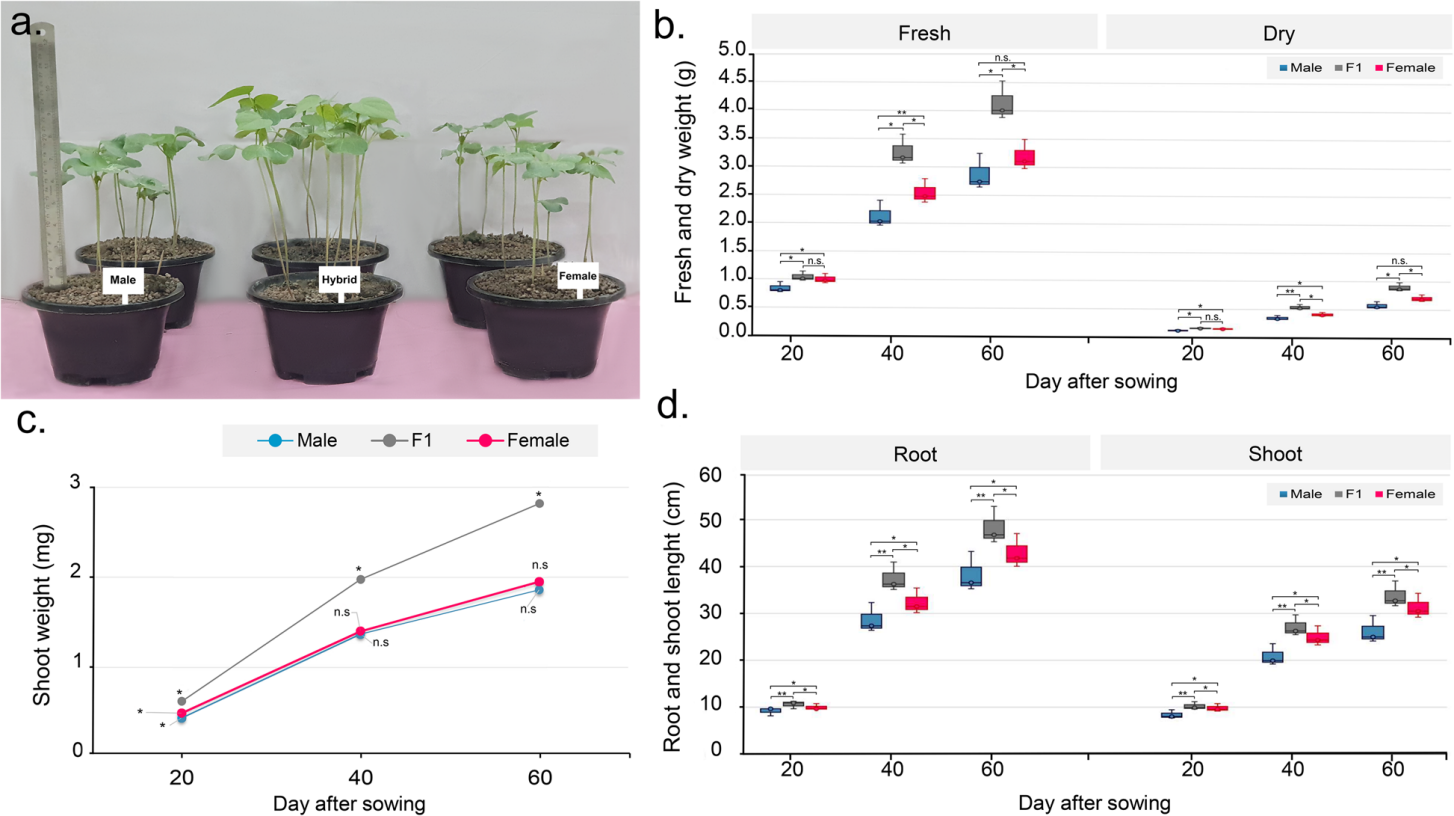
Heterosis performance of hybrid at three different developmental stages. **a** Phenotype of hybrid and its parental lines. **b** Fresh weight and dry weight of the hybrid and its parental lines. **C** Shoot weight of hybrid and its parental lines from day 20 to 60 after sowing (DAS). **d** Shoot and root length of hybrid and its parental lines. Values with different letters are considered statistically significant (shortest significant range; *P* < 0.01)

Moreover, three growth parameters were phenotyped to evaluate the timing of heterosis initiation: plant weight (fresh and dry weight), shoot weight, shoot and root length at three developmental stages: 20 days (seedling stage), 40 days (squaring stage), and 60 days (flowering stage). We compared the MPV with the hybrid. At 20 days, all growth parameters showed a significant increase. However, at 40 days, the hybrid showed higher plant weight (36.7% more than the MPV), shoot height (18.33% more than the MPV), and root length (22.38% more than the MPV) (Fig. 1a-d). Similar results were obtained in the flowering stage. Among the different developmental stages, squaring stages was found to have higher growth in all growth parameters, indicating that the squaring stage has greater heterosis than the seedling and flowering stages, so samples from this stage were selected for downstream investigation (Fig. S1).

### F1 hybrid have more actively expressed genes than its parents

**Fig. 2:**
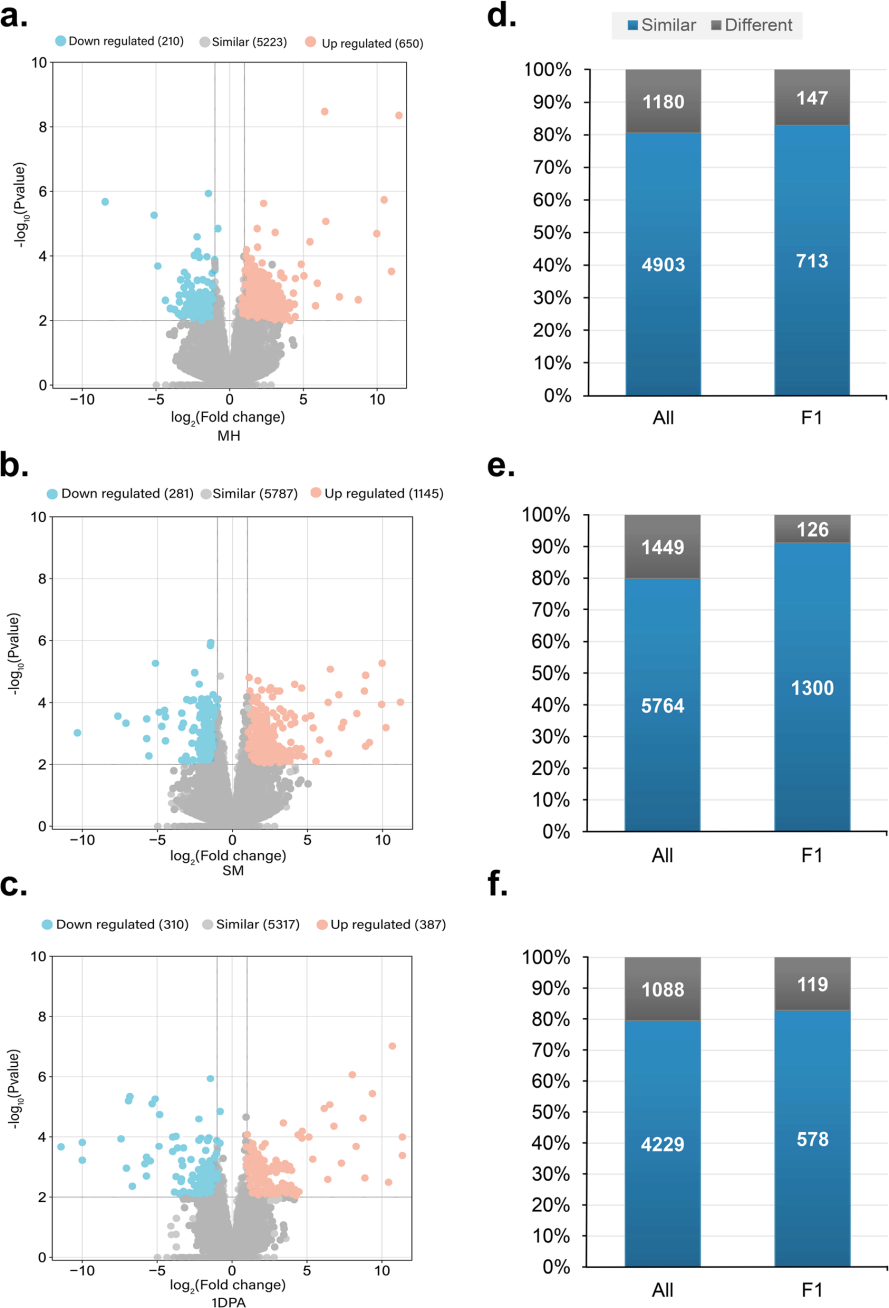
Overview of the genes differentially expressed in the F1 hybrid compared with MPV (mean of the two parental values). **a**–**c** The number of genes differentially expressed in the F1 hybrid compared with the expected MPV (*P* ≤ 0.01; false discovery rate ≤ 0.01) in MH, SM and 1DPA. **d**–**f** Genes that were differentially expressed in the two parents were more frequently associated with differentially expressed genes in the F1 hybrid (χ2 test, *P* ≤ 0.01)

The transcriptomes of F1 and its parents were evaluated for dynamic changes in three biological replicates by assessing transcripts in flower bud samples at the “match-head” (MH), “square growth midpoint” (SM), and one-day post anthesis (1DPA) ovule. Each library generated between 62 and 84 million reads (Table S2). Approximately 94.04% of the reads that were of high-quality, were mapped to the G. *hirsutum* reference genome. In each sample and genotype, approximately 90.84% of reads were mapped to exon regions. Intergenic regions and intron mappings accounted for less than 4.57% of the total (Fig. S2). Principal component analysis (PCA) revealed that the different tissues and genotypes were clearly distinguishable from each other (Fig. S3). In each dataset, around 6400 genes were expressed (Fig. S4). The overall number of expressed genes in square growth midpoint” (SM) developing buds was much greater than in “match-head” (MH) and 1DPA ovules. To identify probable DEGs related to the heterotic phenotype, we compared the transcriptome profile of hybrid with MPV, which we named hybrid MPV-DEGs (Fig. S1, Table S3). It appears that at the three stages, in F1, most genes are expressed additively, with a considerable fraction of genes expressed non-additively (697–1426 or 10–22%) (Fig. 2a-c, & Fig. S5a). MH and SM were found to have more upregulated genes than downregulated genes in F1 (3.1-fold and 4-fold, respectively, χ^2^ test, P ≤ 0.01) (Fig. 2a, b). However, the number of upregulated genes was greater in 1DPA, although the difference was not significant (Fig. 2c).

Furthermore, we examined the DEGs between the two parental lines (termed M-F DEGs) and found that between the two parental lines at each stage, 19.3%, 20.1%, and 20.4% of the total genes were differentially expressed, respectively (|log2 FC|≥2, P-value ≤ 0.01; FDR ≤ 0.01) (Fig. 2d-f). At all three developmental phases, a substantial proportion of these (53 − 89%) overlapped with the MPV-DEGs, implying that genes whose expression levels differed between the hybrid’s parents were overrepresented among those differentially expressed genes in the hybrid (Fig. S5b).

### Importance of high-parental expression level dominance in hybrid performance

**Fig. 3:**
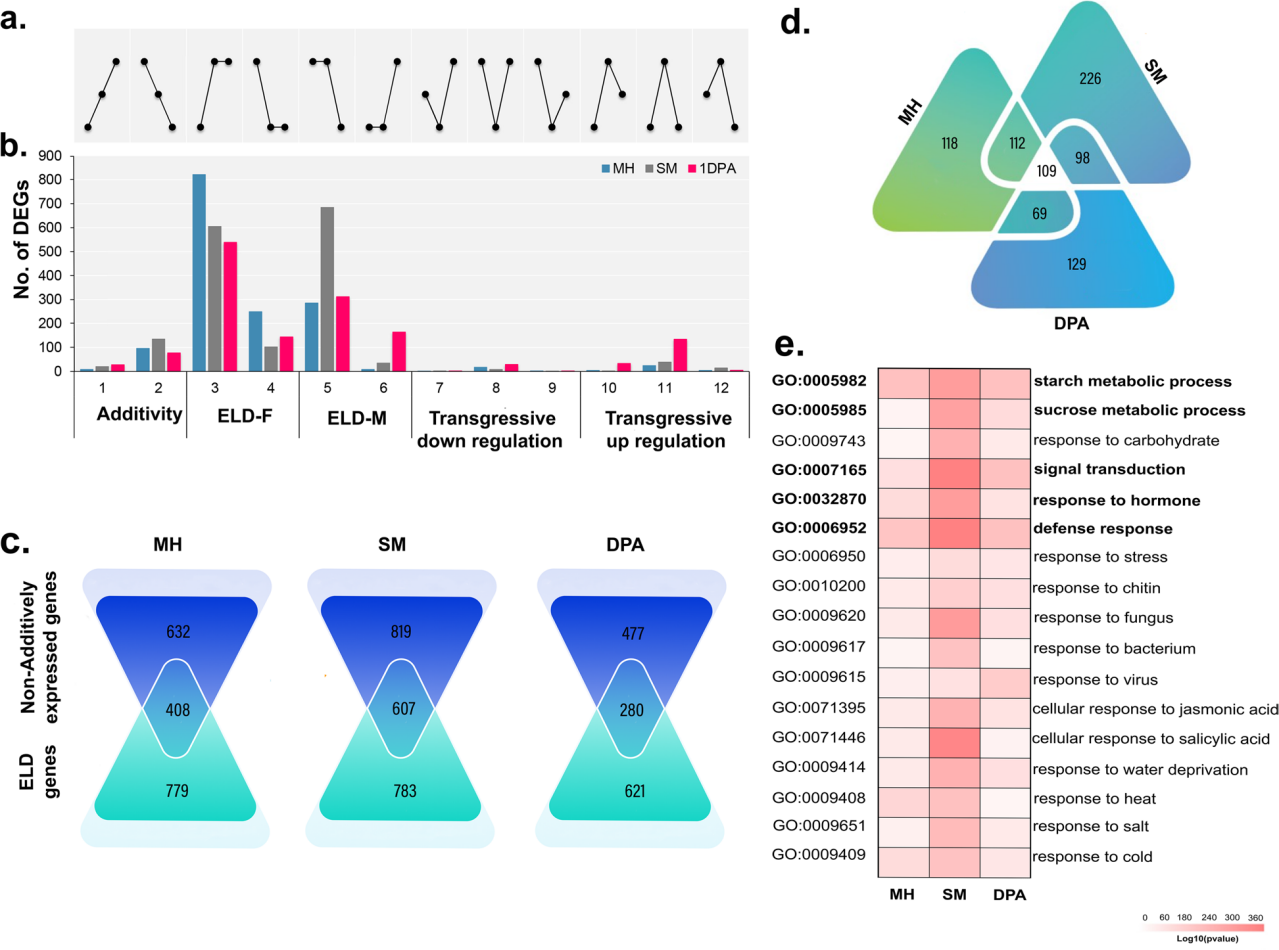
Parental expression level dominance (ELD) genes in the allotetraploid cotton hybrid. Genes with an expression level in the F1 hybrid similar to that of the female parent are designated ELD -F; genes with an expression level in the F1 hybrid similar to that of the male parent are designated ELD -M. **a** Expression patterns of twelve types of differentially expressed genes (DEGs). M, male parent; H, hybrid; F, female parent. **b** Number of genes in each of the 12 types of DEGs in MH, SM, and 1DPA. **c** Venn diagram showing the number of genes that were unique or overlapped in ELDs and DEGs in each tissue. **d** The highly enriched GO terms that are unique or overlapped in all three developmental stages. **e** Highly enriched GO terms of ELD genes

The phenomenon, in which genes with varying expression in parental lines, have expression levels in F1 that are statistically comparable to that of one of the parents, is called Expression Level Dominance (ELD) [21]. ELD has been observed in allopolyploid plants [22]. To determine the amplitude and direction of expression in intraspecific F1 cotton hybrid, DEGs were classified into 12 possible groups (Fig. 3a) [23]. The results revealed that the expression levels of most genes in the hybrid were comparable to the parent. In contrast, just a few genes in these samples are additively or transgressively regulated. Some ELD genes expressed as strongly as the parent with the strongest expression (high parental ELDs; classes 3 and 5 in Fig. 3a), whereas others expressed as weakly as the weakest expressing parent (low parental ELDs; classes 4 and 6 in Fig. 3a). More than 72% (1107/1520), 78% (1290/1648), and 58% (851/1460) of the ELD genes were high parental ELDs in MH, SM, and 1DPA at all three stages, respectively (Fig. 3b). A substantial proportion of MPV-DEGs of the hybrid coincided with genes with high parental ELD (22.42% in MH, 27.47% in SM, and 20.31% in 1DPA, Fig. 3c). These findings imply that high parental ELD genes, in combination with a nonadditive mechanism, may contribute to hybrid heterosis.

To test this hypothesis in more depth, four gene groups (3 to 6 class) were pooled and GO and KEGG (www.kegg.jp/kegg/kegg1.html [24–26]) enrichment analysis was executed to investigate the probable role of ELD genes with high parental content in F1. The highly enriched GO terms overlapped at all three developmental stages [71.25% for MH, 74.4% for SM, and 66.8% for 1DPA, Fig. 3d).], indicating that genes with high parental content ELD may be engaged in comparable biological functions at all three stages. Genes with high parental ELD were observed to be highly enriched in GO terms related to starch metabolic process, sucrose metabolic process, signal transduction, response to hormone, and defense (Fig. 3e).

### F1 hybrid exhibit substantial allelic expression trans-regulation

It is unknown how F1 regulates two different alleles of one gene from different parental lines. The contributions of each parental allele to the overall expression of gene in F1 were distinguished using single nucleotide polymorphisms (SNPs) markers, and the origin and level of expression of the allele in F1 was also determined. Both parents’ genomes were re-sequenced with 165 coverage, and allelic SNPs were identified in the exonic regions of all the annotated genes by mapping their genome sequences to the G. *hirsutum* reference genome. Only the transcripts containing allelic SNPs found both in the re-sequenced genome and the transcriptome were taken into account for the read mapping and expression comparison in order to test the efficacy of parental allelic discrimination.

**Fig. 4:**
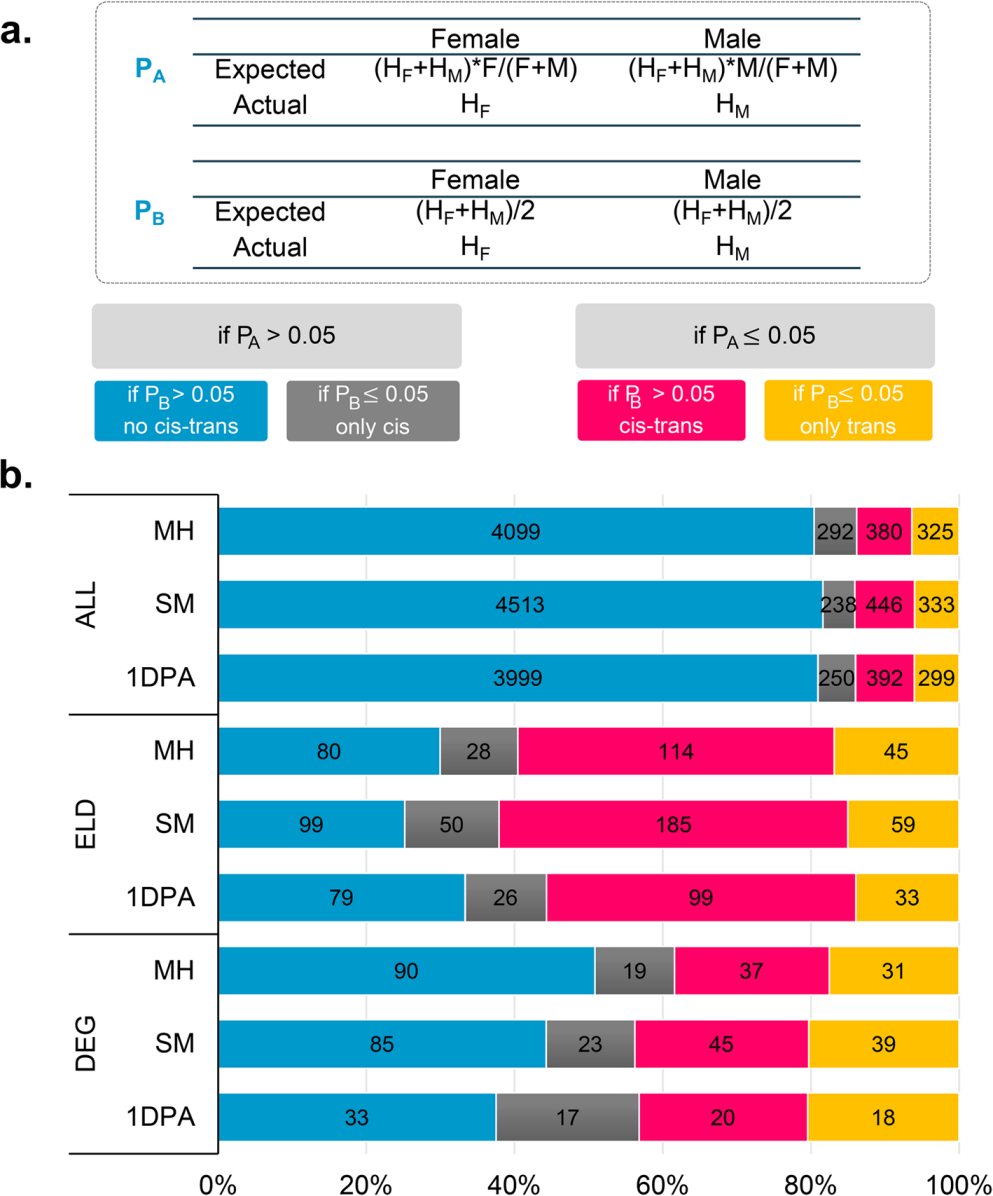
Distribution of genes with cis- and/or trans-effects in MH, SM and 1DPA. **a** Flow chart showing quantification of cis- and trans-effects and classification of the transcripts with cis- and/or trans-effects. F, gene expression level in female parent; M, gene expression level in male parent; HF, female parental allelic expression level in hybrid; HM, male parental allelic expression level in hybrid. **b** Number and percentage of the genes without a cis- and trans-effects, with a cis- or trans-effects only, or with both cis- and trans-effects amongst the expression level dominance (ELD) genes, differentially expressed genes (DEGs) between the F1 hybrid and mid-parent value, and all expressed genes in each tissue

Using the principles described by X Shi et al. [27], we determined whether an allele in F1 was cis- or trans-regulated (Fig. 4a). To assess transregulation, we used *P*-value A (PA, χ2-test) to determine alterations in the expression between the two alleles of the hybrid from the parents and their relative expression patterns in the two parents. If these differences in expression between the parental alleles in F1 differed noticeably from the difference in expression between the two parents, it was assumed that the alleles were controlled by a trans effect (PA ≤ 0.05). The two parental alleles are tested for equivalent expression in the hybrid with a *P*-value of B (PB, χ2-test). If the expression levels of the two parental alleles were significantly different in the hybrid (PB ≤ 0.05), the cis effect was considered to regulate them (Fig. 4a). Based on the test data of PA and PB, four different categories of regulatory effects were identified: trans effects only, cis effects only, cis and trans effects together, and no cis and trans effects (Fig. 4b). Following the elimination of genes which had higher similarity in the sequence between the two parental alleles (i.e., low SNP coverage) or low levels of expression [FPKM < 2], approximately 4950 to 5530 genes with significant allelic expression differences were detected in F1 at all three developmental stages. In MH, 380 (7.5%), 292 (5.0%), and 325 (6.37%) genes were detected as affected by the trans effect only, by the cis effect only, or by both (cis-trans) (Fig. 4b). The remaining 4099 (80.43%) genes showed no differences in allelic expression between parents or F1 progeny (i.e., no cis-trans). The relative pattern of the four categories of regulatory effects in the SM and 1DPA samples was comparable to MH, with a slightly larger proportion of trans effects (5.1–10.5%) than in the MH sample.

Across multiple samples, 897 genes from the ELD genes and 457 genes from the hybrid MPV genes showed significant allelic expression differences in F1. The percentage of ELD genes with only trans effects was significantly greater for MH (42.69%), SM (47.07%), and 1DPA (41.77%) than the all genes (7.4% for MH, 8.6% for SM, and 7.9% for 1DPA) (Fig. 4b). The significance of trans regulation for the ELD genes in the G. *hirsutum* F1 hybrid is revealed by these findings. Cis-trans effects and trans effects both appear to have had an impact on how the hybrid MPV-DEGs functioned. Cis effects had a minimal regulatory effect on the ELD gene and the hybrid MPV-DEGs (Fig. 4b). These findings suggest that trans effects are more significant than cis effects in determining the expression patterns of genes differentially expressed in F1.

To investigate the function of trans-effects in heterosis, we performed GO and KEGG enrichment analyses for all trans-effects in genes in all datasets. Enrichment analysis revealed that trans effects were highly enriched in the GO terms " hormone transport,“ “response to stress,“ and processes related to energy metabolism in all samples (FDR ≤ 0.01) (Fig. S6a). The addition of these factors resulted in a hybrid with better energy performance and tolerance to environmental cues than the parental lines. Moreover, trans-effects were enriched for the terms “biosynthesis of secondary metabolites,“ “nitrogen metabolism,“ and “metabolic pathways” (Fig. S6b). Accordingly, our results suggest that F1 is able to adapt to a variety of environments and promote critical developmental processes by using dominant alleles and expressing them at high levels.

### Lower divergence of homoeologous gene pair expression in the F1 hybrid

**Fig. 5:**
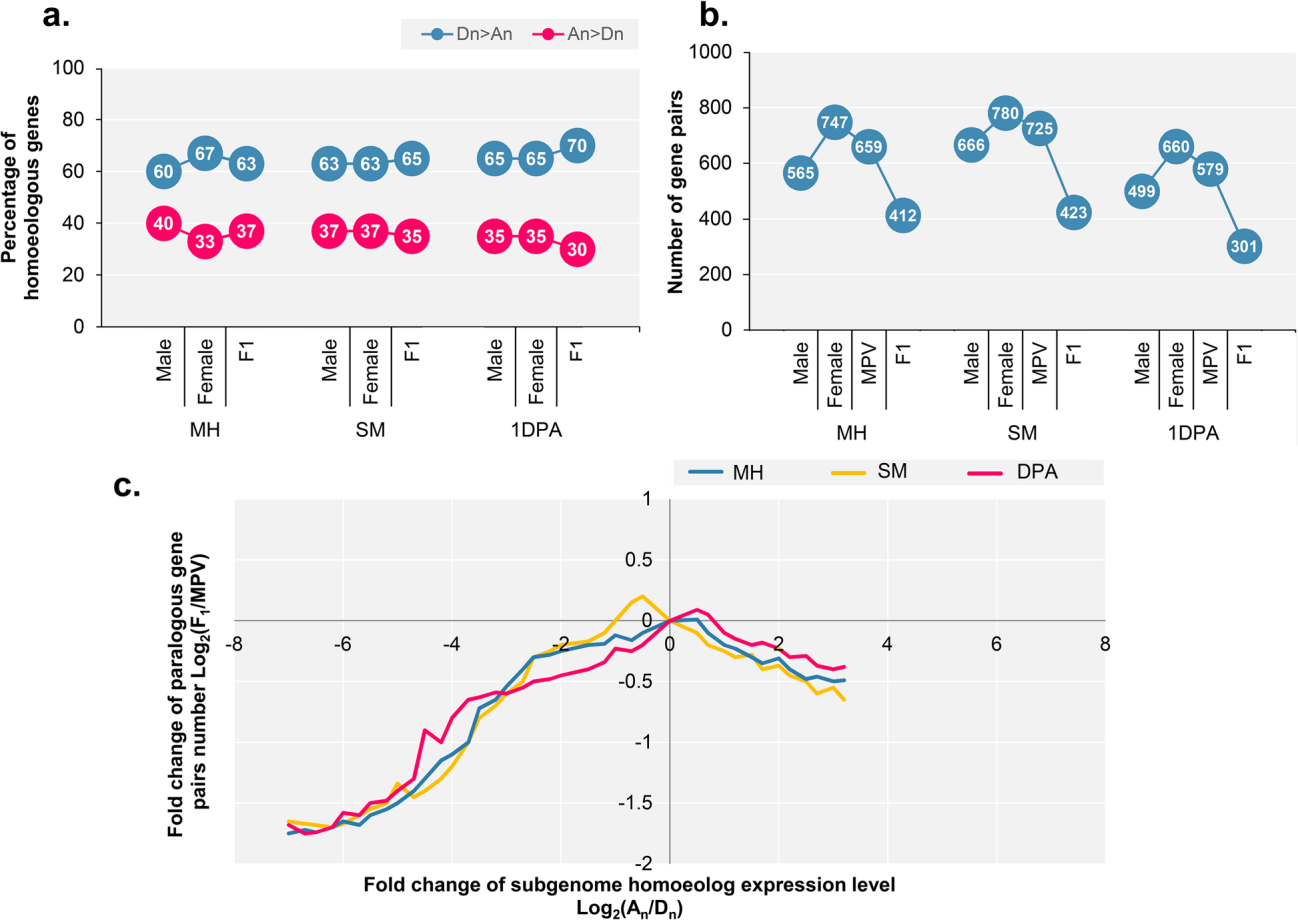
The expression patterns of homoeologous genes of the A_n_ and D_n_ subgenomes in *Gossypium hirsutum.* **a** Percentage of the homoeologous genes with a higher or lower expression level in the D_n_ subgenome than in the A_n_ subgenome. **b** Number of homoeologous gene pairs with a 15-fold (i.e. log_2_(A_n_/D_n_) ≥ 4.0 or ≤ − 4.0) expression difference in the two subgenomes of G. *hirsutum*. MPV, mid-parent value. **c** Distribution of the homoeologous gene pairs based on their expression changes between the F_1_ hybrid and MPV [log_2_(F_1_/MPV)] and their expression difference between the A_n_ and D_n_ homoeologs [log_2_(A_n_/D_n_)] in each tissue

In order to study the extent of homoeologous gene expression in the A_n_ and D_n_ subgenomes of F1 hybrid, first the homoeologous genes expressed in both parents and their F1 hybrid during the three developmental stages were determined. 6044, 7105, and 6033 homoeologous genes were found in MH, SM, and 1DPA, respectively. All samples and genotypes showed stronger expression of D_n_ homoeologous than the corresponding A_n_ homoeologous (i.e., D_n_ > A_n_; Fig. 5a). Next, fold changes in expression levels between homoeologous gene pairs between F1 and its parents were calculated. We observed that in comparison with parents, F1 has fewer DEG pairs. For instance, F1 had a significantly lower number of homoeologous gene pairs with a 15-fold difference in expression [i.e. log2(A_n_/D_n_) ≥ 3.0 or ≤-3.0] at MH (412), SM (423), and 1DPA (301), than that of either parent (Student’s t-test, P ≤ 0.01, Fig. 5b).

In addition, the extent of variation in gene expression between homoeologous gene pairs between parents and their hybrid (MPV and F1) was examined and for most homoeologous gene pairs, F1 had a smaller difference in expression between homologous gene pairs than MPV [i.e., log2 (F1/MPV) ≤ 0)] (Fig. 5c), suggesting that hybridization reduces the divergence of homologous expression. In addition, greater log2 (F1/MPV) values (i.e., diverged more from MPV) were found for homoeologous gene pairs with higher expression in D_n_ subgenome than in A_n_ subgenome (i.e., log2(An/Dn ≤ 0)) than for those that had higher expression in the A_n_ subgenome than in D_n_ subgenome (Fig. 5c). This resulted from the D_n_ subgenome having more highly expressed homoeologous (Fig. 5a). Overall, our findings indicate that following hybridization, variations in the expression of homoeologous in F1 were significantly reduced.

### siRNA profiling, and genomic distribution

The 27 sRNA-seq libraries yielded 213 million reads (48 bp per read), with approximately 6 million distinct sRNAs detected for all three genotypes (Table S4). The 21- and 24-nt classes, according to analysis of the filtered reads’ class distribution, were most abundant in F1 and the two parental lines at all three stages (Fig. S7a). The expression of about 280,000–300,000 clusters of 24-nt siRNA were observed in SM, MH, and 1DPA. At all three stages, most clusters of 24 nt siRNA ranged from 100 to 1000 nt, with 150–250 nt clusters predominant (Fig. S7b).

**Fig. 6:**
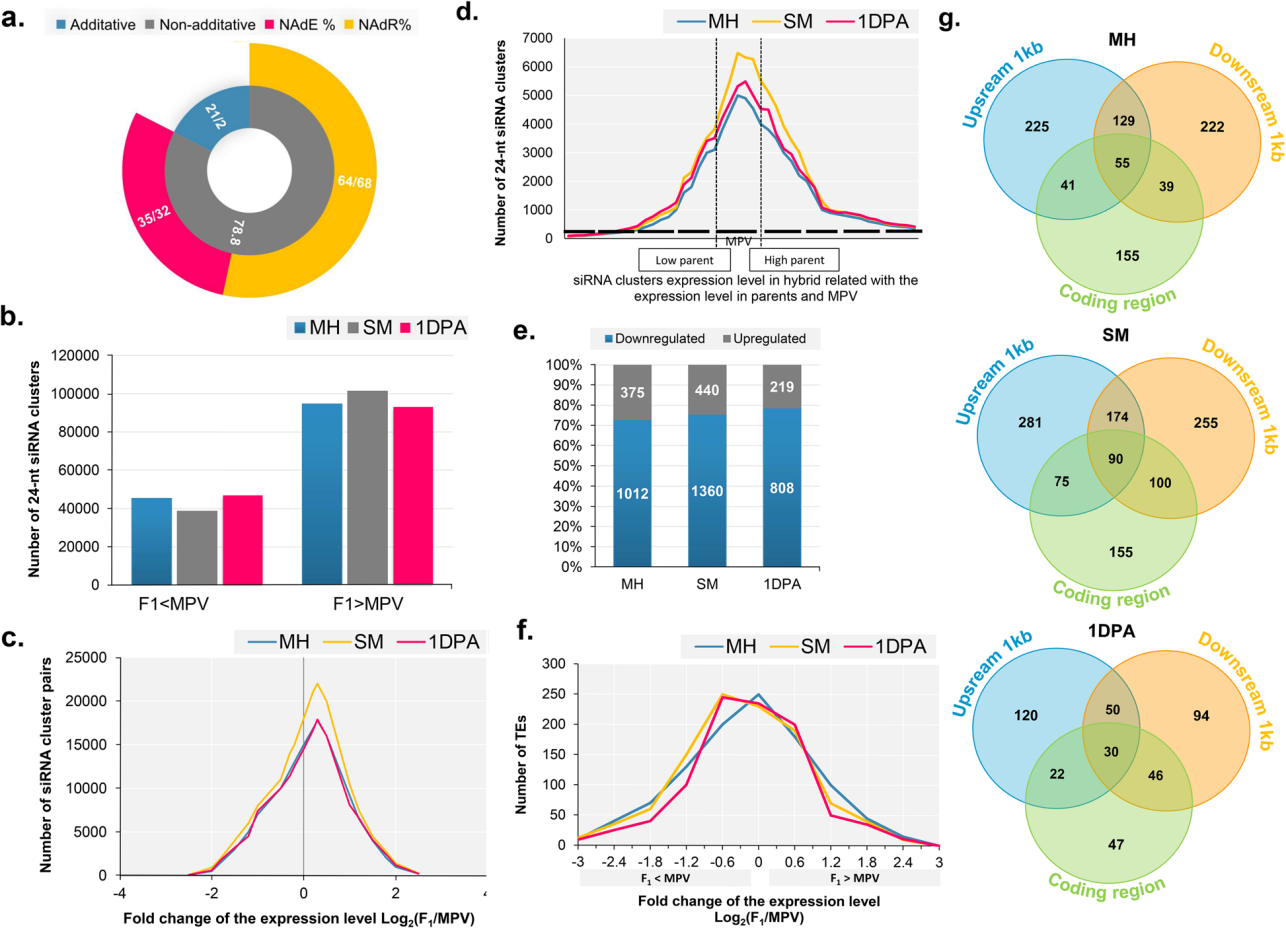
Expression patterns of the 24-nucleotide (nt) siRNA clusters in the F1 hybrid. **a** Non-additive expression of 24-nt siRNA clusters in hybrid compared to MPV. **b** Number of the 24-nt siRNA clusters that have a higher or lower expression level in the F1 hybrid compared with the mid-parent value (MPV). **c** Distribution of the number of the 24-nt siRNA clusters based on their expression level changes between the F1 hybrid and MPV in each tissue. **d** Distribution of the number of the 24-nt siRNA clusters based on their expression levels in the F1 hybrid compared with those in the two parents and MPV. **e** Number of transposable elements (TEs) that were up- or downregulated in the F1 hybrid compared with MPV (fold change > 1.5). **f** Distribution of the number of TEs based on their expression level changes in the F1 hybrid and MPV in each tissue. **g** Venn diagram showing the number of the non-additively expressed genes that had siRNA clusters in coding regions or 1-kb flanking regions in MH, SM and 1DPA.

The differential expression of the 24-nt siRNA clusters in the parental and hybrid line were examined to evaluate the impact of 24-nt siRNA clusters on gene expression. When hybrid and parental lines were compared, it was found that 78.8% of 24-nt siRNA clusters were differentially expressed in F1 and parental lines (Fig. 6a). At all three developmental stages, there were significantly more clusters of 24-nt siRNA with expression levels above MPV (67.6% in MH, 72.4% in SM and 66.5% in 1DPA) than clusters of 24-nt siRNA with expression levels below MPV (32.4% in MH, 27.6% in SM and 33.5% in 1DPA) (paired Student’s t-test, P ≤ 0.001) (Fig. 6b). Considering this, most of the 24-nt siRNA clusters had a log2FC (F1/MPV) value of 0.3 (Fig. 6c), which differed significantly from the null expectation [i.e., F1/MPV log2 (F1/MPV) = 0]. The number of 24-nt siRNA clusters found in F1 and their expression level were plotted on a 2D graph to evaluate the level of expression of 24-nt siRNA clusters in F1 and both parents (MPV) (Fig. 6d). Despite the peak around MPV, the distribution of 24-nt siRNA clusters at all three stages was negatively skewed (skewness > 0), indicating that the expression of 24-nt siRNA clusters in F1 was greater than that in MPV.

The 24-nt siRNA clusters were detected on all chromosomes, and their abundance was examined in three genomic regions: (i) gene-coding regions, (ii) the 2-kb-flanking regions of a gene, and (iii) TEs. More than 74.3% of 24-nt siRNA clusters were colocalized with TEs in both parents and F1 hybrid; nevertheless, the 2-kb-flanking regions had a greater number of 24-nt siRNA clusters than the gene-coding regions (gene related siRNA, 24%) (Fig. S7c, Table S5). Furthermore, the expression patterns of the TEs that gave rise to clusters of 24-nt-siRNA in both parents and F1 were examined to determine whether the transcription of the TEs was affected by the non-additively expressed 24 nt siRNA clusters from the TE. Of the nearly 1127–1800 TEs detected as differentially expressed between MPV and F1 in SM, MH and 1DPA, the number of TEs with lower expression levels in F1 than in MPV was significantly higher than the number of TEs with higher expression levels in MPV (71–75%, Student’s t-test, P ≤ 0.01; Fig. 6e). Considering this result, more than half of the TEs that produced 24-nt siRNAs, had log2(F1/MPV) values between − 0.2 and − 0.6, which differed from the predicted log2(F1/MPV) value of 0 (see Fig. 6f). This indicates that in F1, the TEs’ activity related to the 24-nt siRNA clusters was reduced.

According to the distribution study, most of the 24-nt siRNA clusters associated with genes were detected in 2-kb regions upstream or downstream of non-additively expressed genes. siRNA clusters were found in 2-kb upstream regions in 450, 620 and 222 genes; in 2-kb downstream regions in 445, 619 and 220 in the hybrid MPV-DEGs; and in coding regions in 290, 420 and 145 genes in MH, SM and 1DPA, respectively (Fig. 6g). In addition, the 24-nt siRNA clusters are differentially expressed in 335 (28%), 641 (38%), and 250 (42%) non-additively expressed genes in the hybrid MPV-DEGs in MH, SM, and 1DPA, respectively (Table S5).

### MiRNAs are non-additively repressed more in the F1 hybrid

**Fig. 7:**
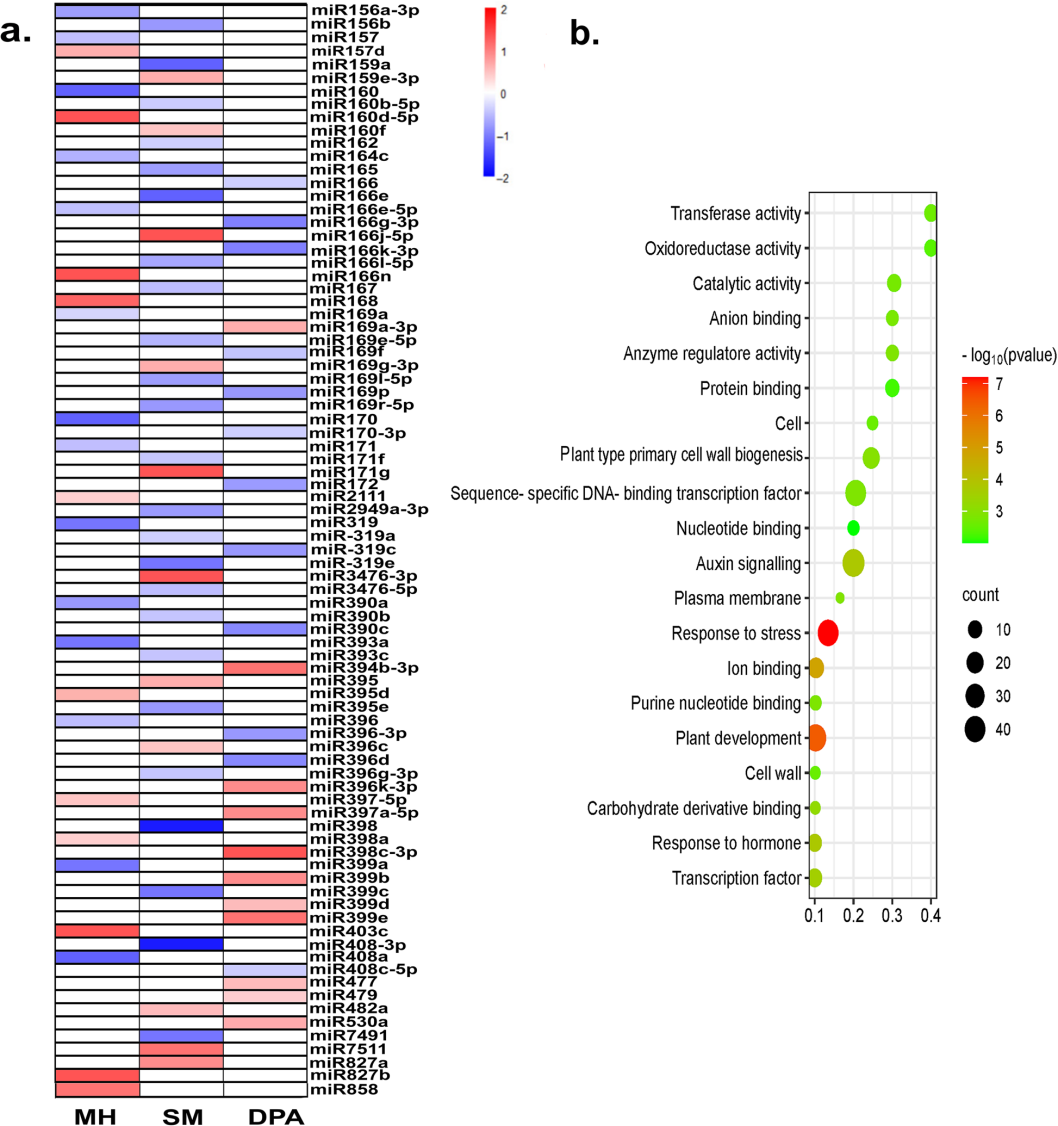
Changes of miRNAs and their targets in the F1 hybrid. **a** Expression changes of the differentially expressed conserved miRNAs in the F1 hybrid compared with the mid-parent value (MPV) in the three samples. **b** Significantly enriched Gene Ontology terms of the differentially suppressed miRNAs targets in the F1 hybrid

To examine the possible contribution of miRNA to the heterosis in G. *hirsutum*, the expression patterns of hybrid and both its parents were analyzed at three developmental stages. In total, 105 known miRNAs from 65 families were found in three genotypes. At the three stages, 83 (38 families) of the 105 conserved miRNAs were identified as not additively expressed between hybrid and parental lines (P ≤ 0.01, FDR ≤ 0.05, Table S6a). Out of the 83 differentially expressed miRNAs, there was non-additive repression of 14, 24, and 12 miRNAs in MH, SM, and 1DPA, respectively, whereas non-additive activation of 11, 10, and 12 miRNAs in MH, SM, and 1DPA, respectively. Some of the best functionally characterized miRNAs conserved between monocot and eudicot were found in this category (for example, miR159/160, miR164/166, miR319, miR393, miR396, and miR408) (Fig. 7a). Over and above the known miRNAs, 32, 46, and 34 novel miRNAs were found in MH, SM, and 1DPA, respectively (Table S6a). Based on their expression levels in F1 and parental lines, we found that F1 had more down-regulated novel miRNAs than up-regulated novel miRNAs in MH (21/32, 65.25%), SM (34/46, 73.91%) and 1DPA (24/34, 70.58%).

Using gene models from *G*. *hirsutum* genome annotation [28, 29], and psRNAtarget analysis, we could predict the target genes of these non-additively expressed miRNAs (DEmiRs) [30]. 9789 targets were found, including 4961 for 83 known miRNAs and 4828 for 112 novel miRNAs (Table S6b&c). The majority of miRNAs processed a wide range of target gene families, including MAP kinase, UDP Glycosyltransferase superfamily protein, Expansin, growth-regulating factors (GRFs), GRAS family transcription factor, Starch synthase 2, calcium-dependent protein kinase, and Ethylene response factors. The correlations between targets and miRNAs were assessed using the Pearson correlation coefficient approach [31]. A strong negative correlation (r = 0.78) was observed between the expression levels of the known miRNAs and their corresponding target genes. To better understand the likely involvement of suppressed miRNAs in heterosis, their target genes were subjected to GO enrichment analysis. Most of the target genes were functionally annotated in eight biological processes, nine molecular components, and four cellular components, including auxin signalling, plant development, stress response, sequence-specific DNA-binding transcription factor, and ion binding (P ≤ 0.01) (Fig. 7b).

### Global methylation profile of hybrid and its parental lines

To investigate the role of epigenetic regulation in heterosis, we used bisulfite sequencing to create maps of methylated cytosines at single-base resolution at the SM stage of the hybrid and its parental lines. A total of 1641 million (about 268 million per sample) clean bisulfite sequencing reads with an average length of 150 bp were gained from the parental lines and their hybrid. 82.25% of a total read, were uniquely mapped to the reference genome of G. *hirsutum*, representing more than 10-fold coverage (Table S7a). Based on mapping efficiency of 0.4–0.6% against the chloroplast DNA reference genome coverage, samples from both parents and hybrid had > 99% bisulfite conversion efficiency in replicates (Table S7b). PCA showed that the different genotypes could be clearly distinguished from each other (Fig. S8a). Pearson correlation analysis was employed to test the reproducibility of the results, and a high correlation (0.92–0.96) was observed between the biological replicates of each sample (Fig. S8b).

**Fig. 8:**
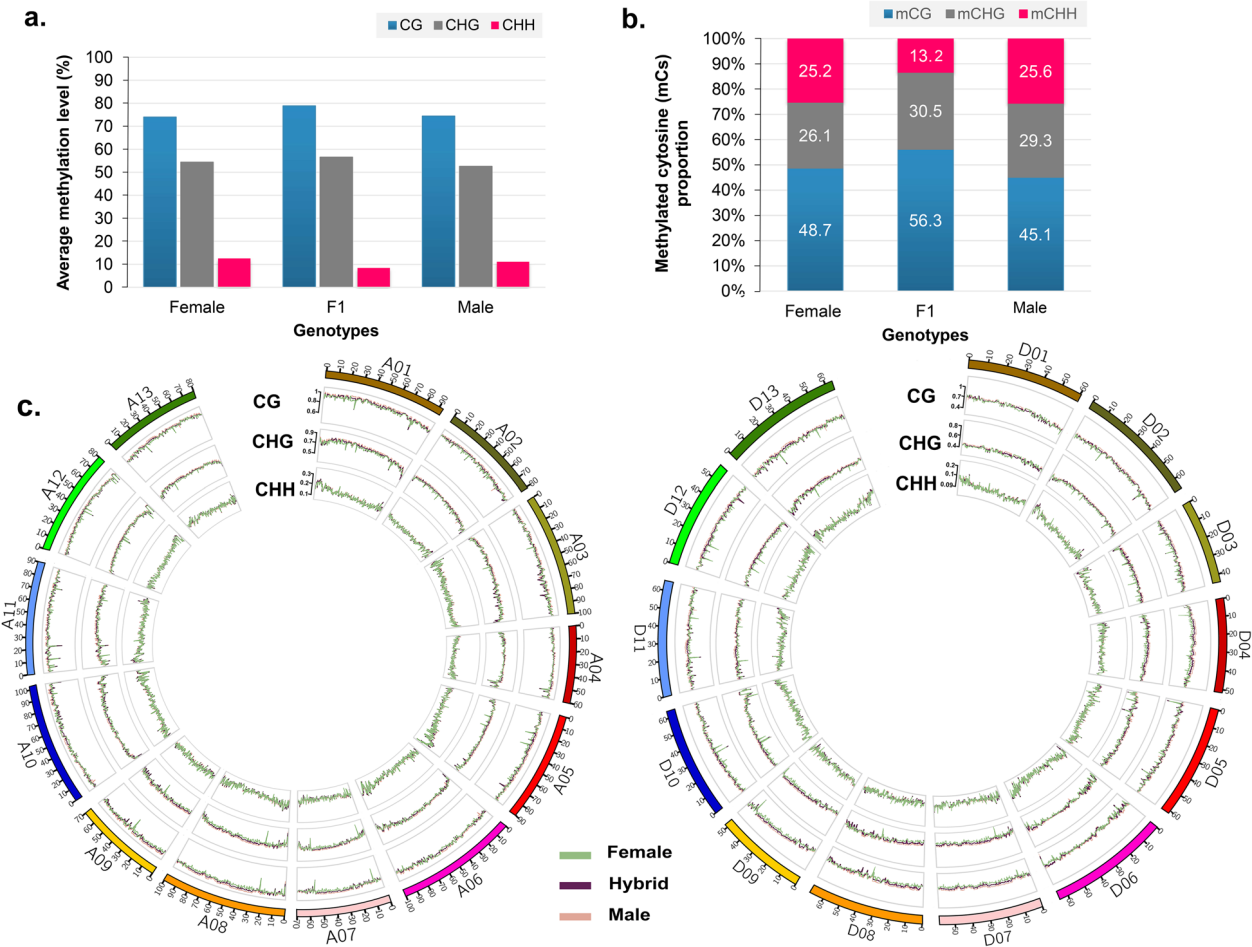
Global methylome maps and DNA methylation landscapes of hybrid and parental lines. **a** Average methylation, (**b**) methylated cytosine (mCs) proportion, and (**c**) average methylation distribution across 26 G. *hirsutum* at CG, CHG, and CHH methylation context

The dynamics of cytosine methylation at CG, CHG, and in the CHH context vary genome-wide between the parental lines and their hybrid. In comparison to either parent, the hybrid displayed lower average methylation in the CHH context and higher average methylation in the CG and CHG contexts (Fig. 8a). In addition, methylated cytosine frequencies in the F1 hybrid and its parental lines ranged from 33.67 to 37.17% (Table S7c). The proportion of mCs in three genotypes was approximately 48.2–56.3%, 26.1–30.5%, and 13.3–25.6% in CG (CpG), CHG, and CHH respectively (Fig. 8b). Of note, the hybrid had a 1.18-fold higher mCs content (56.3 and 30.45%, respectively) in the CG and CHG contexts but a 1.98-fold lower mCs content in the CHH methylation context compared with both parents (Fig. 8b). Average methylation across all 26 chromosomes in all three genotypes was higher in the CG and CHG contexts than in the CHH context, according to chromosome-by-chromosome segmentation of average methylation (Fig. 8c). In addition, the hybrid and male parent had higher methylation than the female parent in chromosomal regions chrD08, chrA07, and chrA09. However, compared to the male, the hybrid’s methylation of the same chromosomes was slightly lower (Fig. 8c).

The parental lines and their hybrid were tested for the mCs’ pattern in several genomic regions (Fig. Sa-f). Approximately 40% of these mCs sites were found to be gene-related, whereas 60% were found associated with transposon elements (TEs). The methylation pattern within the bodies of transposable elements (TE-bodies) and their 2 kb flanking regions for DNA elements, long terminal repeats (LTRs; copia and gypsy), and non-LTRs (LINEs and SINEs) was examined (Fig. S9a-c). For all TE types in TE body sequence, the hybrid showed higher levels of methylation than both parents in all methylation contexts (Fig. S9b-c). For DNA elements and non-LTRs, the methylation pattern was similar in all contexts, i.e. the methylation level was preferentially higher at the TE body than in the flanking regions (Fig. S9 a &c). In contrast, for LTRs, the methylation pattern was different in the different contexts: in the CG context, the TE body had higher methylation levels than the flanking regions, whereas in the CGH and CHH contexts, the flanking regions had higher levels of methylation (Fig. S9b).

In addition, mCs within genes body or within 2 kb flanking regions were also examined. The average gene body methylation (GBM) was highest in the CG context, followed by the CHG and CHH contexts, for both the hybrid and its parental lines (Fig. S9d).The degree of gene body methylation (GBM) in the CG and CHG contexts resembled the 2 kb flanking area and was highest in the hybrid, followed by the female and male parents. The GBM in the CHH context had significantly lower methylation than the 2 kb flanking regions. Remarkably, the hybrid had the lowest average GBM in the CHH context; however, the methylation frequencies in both paternal lines were comparable (Fig. S9d). To avoid potential noise from transposable elements, the GBM was divided into exonic and intronic regions. Methylation of the exonic body was lower than that of the flanking regions, whereas methylation of the intronic body was higher than that of the flanking regions in all contexts in the three genotypes (Fig. S9e&f). When we compared the methylations in the hybrid and the parents in three different contexts, we found that the hybrid in the exonic body had lower methylation levels than its parents. In the intronic body, the methylation level of the hybrid genome followed the same pattern, except in the CHH context (Fig. S9e&f).

### Differentially methylated regions analysis in hybrid and its parental lines

**Fig. 9:**
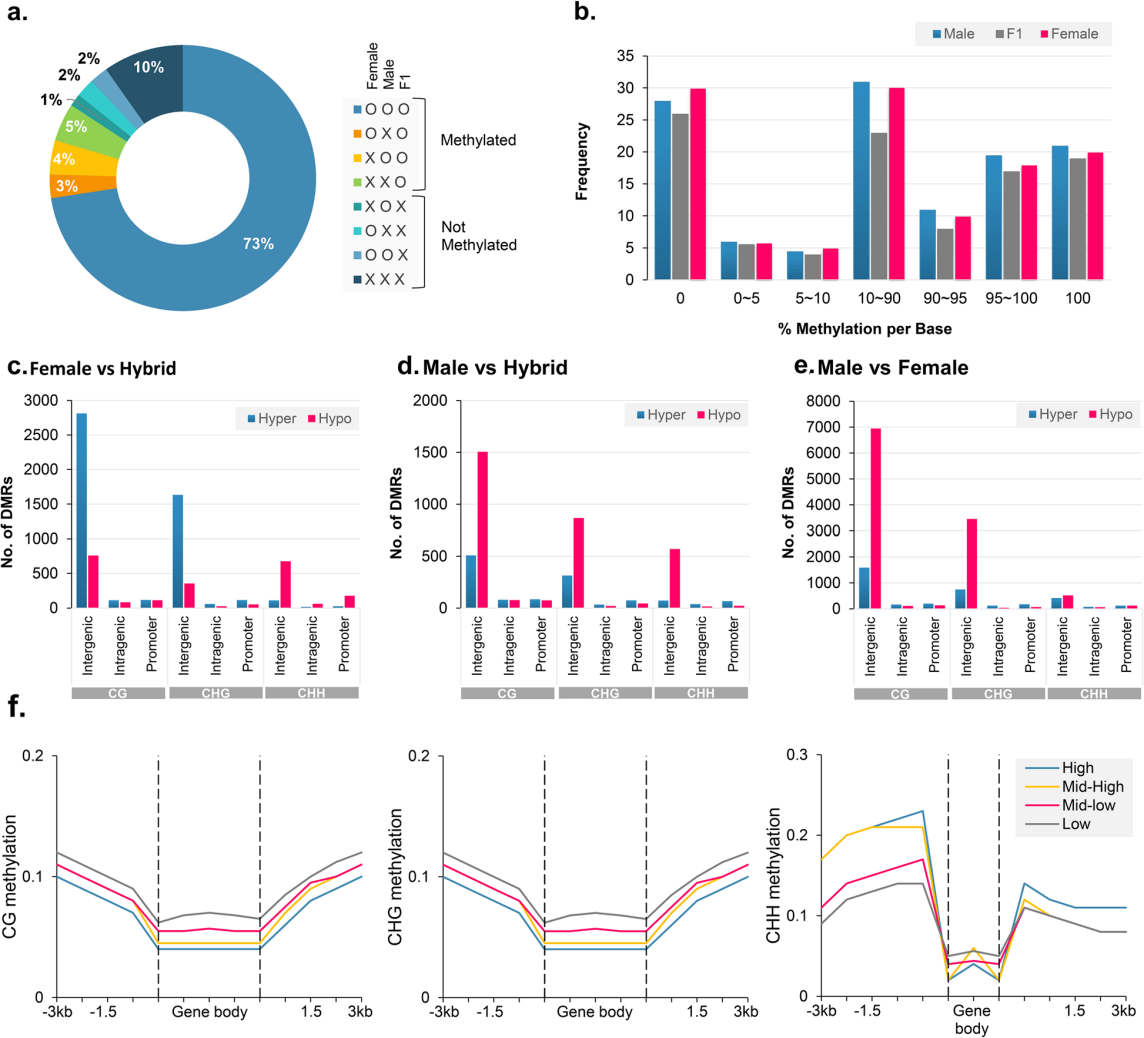
Methylation patterns in the two parents and their F1 hybrid. **a** Distribution (by per cent of total) of the eight methylation patterns. **b** Histogram showing the distribution frequency of the cytosine methylation level. **c**-**e** Bar plots representing DMRs distribution at intergenic, intragenic, and promoter regions in (**c**) Female vs. hybrid, (**b**) Male vs. hybrid, and (**c**) Male vs. Female. **f** Relationships between CG (left), CHG (middle) and CHH (right) methylation and expression levels of genes, which were divided into four quartiles (high, blue; mid-high, yellow; mid-low, pink; and low, gray)

A total of 15,488, 8199, and 3603 unique DMRs were identified in the methylation contexts CG, CHG, and CHH, respectively, by analysing the differentially methylated regions (DMRs) between the hybrid and its parental lines (female, male) (Table S8). Methylation levels varied significantly (either higher or lower) from MPVs in 94.25% (27,289) of the detected DMRs, indicating a nonadditive methylation interaction (interactive DMRs (I)) in these regions (Table S8). In contrast, a limited number (5.75%) of DMRs were categorized as noninteractive (NI), indicating that they were present in an additive manner (*P*-value ≤ 0.001). Differentially methylated regions were divided into transchromosomal methylation (TCM), in which hybrid had significantly higher methylation levels than MPVs, and transchromosomal demethylation (TCdM), in which hybrid had significantly lower methylation levels (FDR ≤ 0.01). Of the 27,289 DMRs identified in the hybrid, there were 14,402 TCM DMRs and 12,887 TCdM DMRs (Table S8). Comparison of the percentage of methylation contributed by the parental lines in the hybrid revealed that 73% of the loci in the hybrid were methylated by both parents. It was observed that Latif and Taban uniquely each contributed 2.92% and 4.26% of the methylation in the hybrid, respectively (Fig. 9a). Interestingly 4.72% of loci had no methylation in the parents but were methylated in the hybrid (de novo methylation). This might explain why F1 has a higher degree of methylation. The frequency distribution of methylation levels of individual bases shows that there was no change in the number of highly methylated cytosines, but there was a decrease in the unmethylated cytosines in F1 (Fig. 9b), indicating that the decrease in unmethylated cytosines was mainly responsible for the increase in methylation levels in F1. Furthermore, analysis of the genomic distribution of DMRs revealed that the majority of DMRs in the hybrid and its parents originated from intergenic regions of the genome (Fig. 9c–e). DMRs from intergenic, intragenic, and promoter areas accounted for about 82.2-86.84%, 4.2–4.8%, and 6.8–8.2% of total DMRs in female versus hybrid, respectively (Fig. 9c). Similarly, in male vs. hybrid, of total DMRs, 80.6–85.7%, 3.8–8.2%, and 4.9–8.2% were from intergenic, intragenic, and promoter regions, respectively (Fig. 9d). Also, comparing female vs. hybrid, all intergenic, intragenic, and promoter DMRs were hyper-methylated in the CG and CHG methylation contexts, but hypo-methylated in the CHH methylation context (Fig. 9c). In male vs. hybrid and male vs. female, intergenic DMRs were hypo-methylated in all the three methylation contexts (Fig. 9d, e).

In addition, we examined the methylation level in DEGs and found that of the 1912 nonadditively expressed genes in F1, 925 (74%), 946 (75%), and 748 (59%) had different levels of DNA methylation in 1 kb upstream, 1 kb downstream, and coding regions, respectively, which we termed differentially methylated genes (DMGs). We categorized DMGs into four groups according to their level of expression (from low to high) and compared them with DNA methylation alterations so as to further examine the connection between gene expression and methylation level. The methylation level in the gene body was found to be inversely related to the gene expression level (in all three contexts), but genes with different CHH methylation level in the upstream region had the highest expression level, proving that altered DNA methylation levels affect the gene expression level in the hybrid (Fig. 9f).

### Higher levels of DNA methylation in hybrid are associated with the 24-nt siRNA clusters

**Fig. 10:**
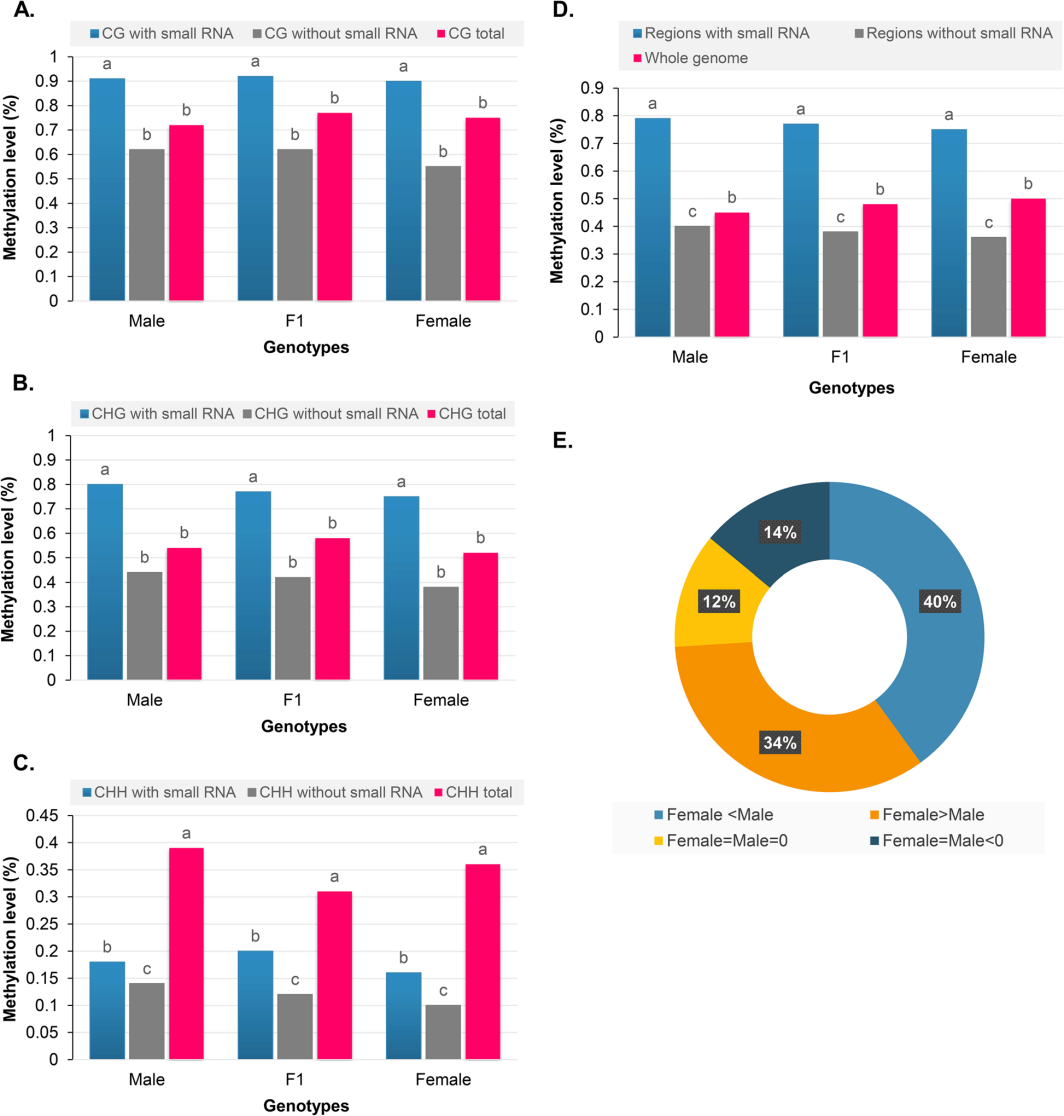
The genomic distribution of 24-nt siRNAs clusters in hybrid and its expression. Regions covered by 24-nt siRNA account for most of the methylation increase in CG (**a**), CHG (**b**), and CHH (**c**) cytosine contexts in hybrid. **d** Contribution to increased DNA methylation in F1 hybrid by the regions with and without 24-nt siRNA in comparison with the whole genome. Duncan’s analysis was employed to test statistical significance. Different alphabets indicated in the graphs revealed significant differences between the groups at *P* < 0.05 level of significance. **e** Contribution to increased DNA methylation in F1 hybrid by the regions with or without 24-nt siRNA. The cytosine positions of the genome were divided into four categories based on the methylation levels of the parental lines (i) positions highly methylated in Maternal line than paternal line (Female > Male), (ii) positions highly methylated in paternal line than Maternal line (Male < Female), (iii) positions where methylation was detected but levels were equal in Maternal line and paternal line (Female = Male > 0) and (iv) positions lacking detectable methylation in both Maternal and paternal lines (Female = Male = 0)

siRNAs can induce DNA methylation at loci with homologous sequences in plants through a process known as RNA-directed DNA methylation (RdDM). To investigate the involvement of this process in the epigenic regulation of heterosis, we analysed the probable association between DNA methylation and 24-nt siRNA using our data. The results showed that siRNA-containing regions in hybrid and parental lines had significantly higher DNA methylation levels than non-siRNA-containing regions in all contexts (Fig. 10a-d), suggesting that the RdDM pathway influences a substantial portion of the methylation sites at these loci. To determine whether siRNA is involved in hybrid-methylome interactions or not, we searched for clusters of 24-nt siRNA in the TCdM and TCM DMRs. We found clusters of 24-nt siRNA in 75.88% of hybrid TCM DMRs and 79.63% of hybrid TCdM DMRs (Table S9). The presence of 24-nt siRNA clusters at different methylation sites was not randomly distributed and followed a particular pattern, namely more siRNAs at CHH methylation sites followed by CG and CHG (Table S9). In the hybrid, 74.34% (TCM) and 79.65% (TCdM) of the clusters of 24-nt siRNA were equally contributed by both the parents. Approximately 3–6% of the clusters of 24-nt siRNA of the parents were exclusive, whereas 42–46% of the clusters of 24-nt siRNA of the hybrid were from the parents (Table S9). In addition, there were some DMR regions (~ 2-5% in the hybrid) in which clusters of 24-nt siRNA were present only in the hybrid but not in the parental lines. These 24-nt siRNA clusters in the hybrid line could be the result of trans generation of a parent that led to DNA methylation in the hybrid line. Categorisation of the small 24-nt-siRNA clusters based on the level of methylation in the parental lines also revealed that the 24-nt-siRNA clusters containing regions that were differentially methylated in the parental lines accounted for 74% of the increase in methylation in the hybrid line (Fig. 10e).

We also investigated the connection between 24-nt-siRNA clusters, methylation, and DMG expression levels. The results showed that CHH methylation on rich-siRNA regions was significantly lower in F1 than in the parental lines (Fig. S10). This result, indicating that siRNA abundance does not positively correlate with levels of DNA methylation in the CHH context. The ability of 24-nt siRNA clusters to move and thus facilitate epigenetic regulation makes them an attractive possibility for regulating transgressive phenotypes in hybrid.

### Trade-off between growth vigour and plant-pathogen interaction

**Fig. 11:**
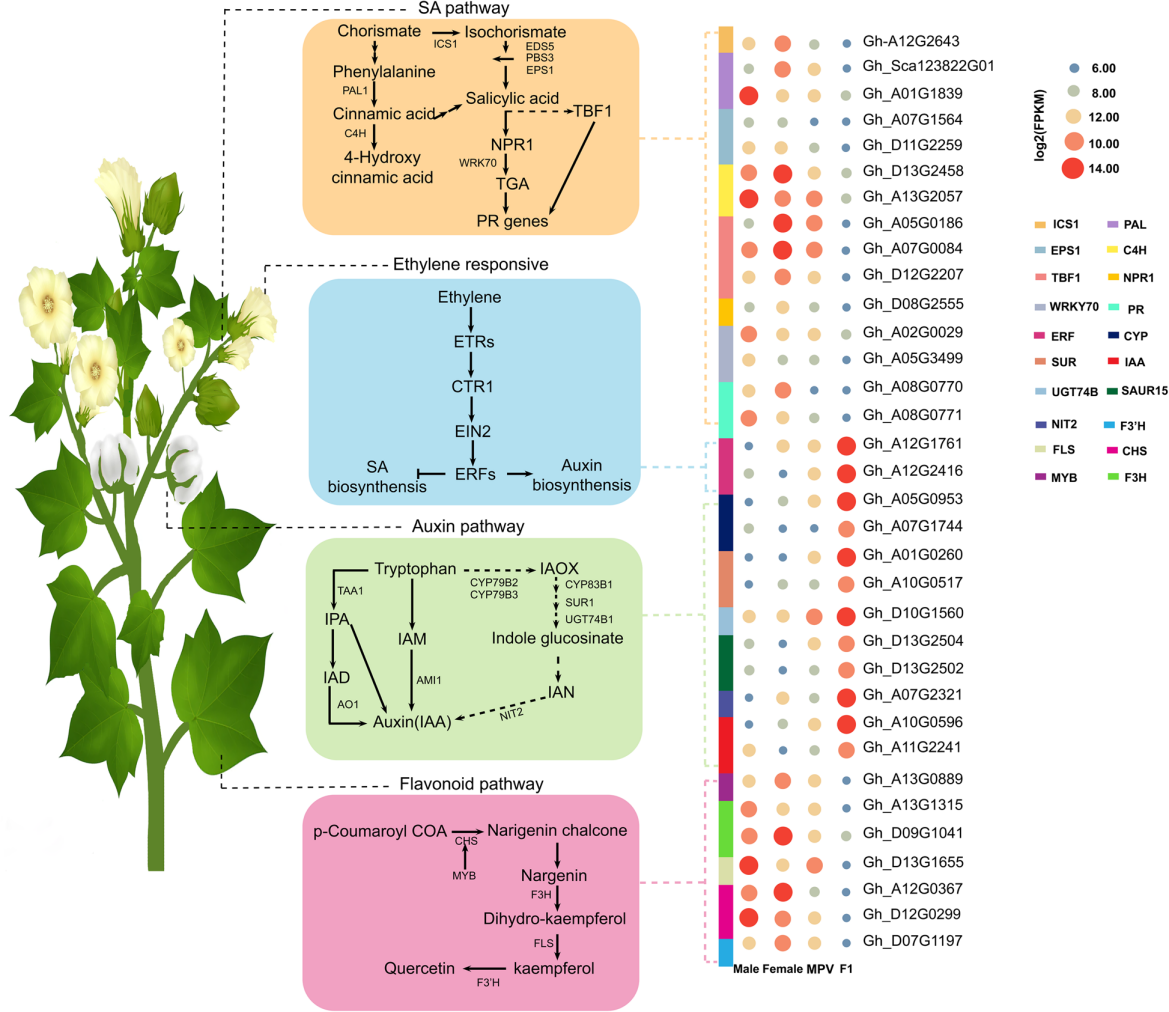
Role of differentially expressed genes (DEGs) and differentially methylated genes (DMGs: methylated-hybrid-MPV DEGs) in the hybrid. DEG Patterns in several key regulatory networks associated with heterosis, including plant growth (auxin and flavonoid pathways), defence (SA pathway and flavonoid pathway), and stress response (SA pathway and ethylene pathway), are presented for the hybrid. Suppressed expression of methylated genes results in altered response in defence, SA and flavonoid pathway. Heatmap showing log2 (fold change) of altered expression patterns of genes involved in the auxin biosynthesis pathway SA pathway, ethylene pathway, and flavonoid pathway. Gene names are colour coded to show the change in expression between hybrid

To comprehend the molecular mechanism underlying allotetraploid cotton heterosis, we performed GO and KEGG enrichment analyses for the MPV-DEGs. The enrichment analyses revealed 150 significantly enriched GO terms (Fisher’s exact test P ≤ 0.01; FDR ≤ 0.05) (Table S10, Fig. 11). Among the different sub-ontologies (BP, MF, CC), Biological Process (BP) had the highest number of significant terms (FDR < 0.05). The significantly enriched biological categories over these lists of GO terms were clustered using REViGO. The most notable functional clusters that moved to F1 were “Plant-pathogen interaction” (1075 genes), “Biosynthetic process of plant hormones and signal transduction” (869 genes) and, “Regulation of transcription-DNA-directed” (676 genes). These clusters, accounting for more than half of the biological terms (FDR < 0.05), might be related to the observed heterosis (Table S10).

Plant-pathogen interaction” in F1 hybrid was one of the most enriched biological terms. Notably, there was a down-regulation in several known pathogen response genes in the F1 hybrid, including R genes (Gh-D05G3723), F-box protein (Gh-A01G0235), MLO-like protein (Gh-A02G0615), Pathogenesis Related Gene 1 (PR1, Gh-A05G0022), chitinase-3-like protein (Gh-A04G1304), disease resistance protein (RPS2, Gh-A03G2102), and Lipid Transfer Protein 3 (LTP3, Gh-A02G0264). There is a close relationship between SA and plant defense responses. A wide range of genes induced by SA, from pathogenesis genes to WRKY transcription factors, were down-regulated in F1 (Fig. 11). The decrease of SA and expression of relevant genes for defense mechanism in F1 hybrid could release more resources for plant growth. TBF1 (Tl1 Binding Transcription Factor1) is a transcription factor activated by SA that affects the allocation of resources between defense and growth. TBF1 inhibits genes involved in plant growth, such as chloroplast proteins, while it increases the expression of genes involved in plant defense. TBF1 expression was found to be more than five times lower in F1 hybrid than in MPV. The parental lines showed either down-regulation (maternal) or up-regulation (paternal) of TBF1 and a number of TBF1-controlled genes. The decreased expression of TBF1, like that of other SA-responsive genes (Fig. 11), was mainly due to a substantial decrease in expression of the maternal allele. Chloroplast-associated genes, one of TBF1’s downstream targets, were upregulated, whereas TBF1-induced defense genes were downregulated, mainly by alteration in the maternal allele expression (Table S3). Similarly, several Ethylene Response Factors (ERF) (SA-repressed genes) involved in plant growth and response to abiotic stress, including as ERF114, ERF112, ERF117, and ERF118, were unregulated in the hybrid (Fig. 11). The altered expression pattern of defense-related genes observed in F1 indicates that the basic defense system of hybrid may be reduced. The reduced activity of hybrid defense cells could be a critical component for improved growth if we consider the opposed association between plant development and plant immunity.

The growth and development of plants are regulated by hormones. According to enrichment analysis, the most important hormone associated with the term “plant hormone signal transduction” is auxin (Indoleacetic Acid (IAA)). Although no genes for main auxin biosynthesis pathway were detected in the DEGs of the MPV hybrid, the auxin transporter protein (AUX1) and genes responsive to auxin (SAUR, IAA17, and NIT2) were significantly enriched and upregulated in the F1 hybrid, suggesting that the rapid response of the hybrid to auxin may stimulate plant growth (Fig. 11). Over and above the changes in auxin biosynthesis, changes in auxin transport affecting local auxin concentrations could also lead to variations in auxin responsiveness. Flavonoids are repressors of auxin transport [32], and the hybrid exhibited down-regulation of flavonoid biosynthesis genes, reflecting a decrease in the level of flavonoids in the hybrid and possibly increased IAA transport rates compared with the parental lines. The downregulation of flavonoid production has far-reaching consequences for the transcriptome of the hybrid, as shown by the overrepresentation of genes regulating flavonoids genes in an enrichment pattern associated with a drop in flavonoid concentration (Fig. 11). These impacts of lower synthesis of flavonoid on the transcriptome of the hybrid seem to be facilitated in part by changes in localized concentrations of IAA, as evidenced by the fact that more than half (60%) of flavonoid-regulated differentially expressed F1 genes are also controlled by auxin. These coregulated genes include those that are extremely sensitive to variations in the concentration of auxin (i.e., typical auxin-inducible target genes). Among the auxin-independent flavonoid-responsive genes discovered in the GO analysis are those associated with metabolism of glucose, transport and responses to abiotic stresses (Fig. 11). Despite improvement in the transport of auxin, variation in flavonoid production accounted for only 24% of the differentially expressed F1 genes regulated by auxin, suggesting that changes in biosynthesis of auxin were the main mechanism impacting auxin responsiveness in hybrid.

**Fig. 12:**
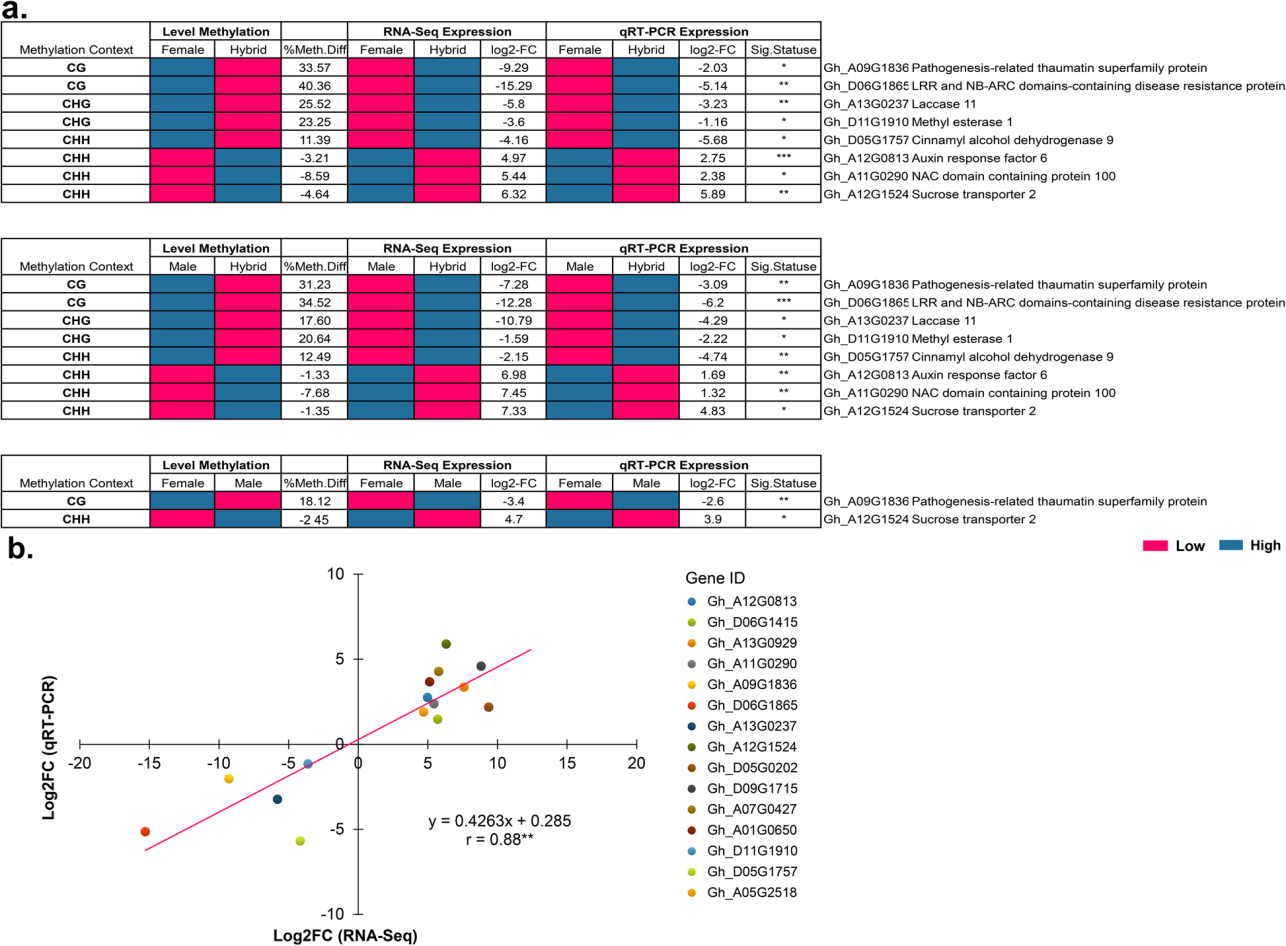
Gene expression observed in quantitative real-time (qRT) PCR analysis. **a** Eight (8) significantly (*p*-value < 0.01) differentially expressed genes (DE-Gs) showing negative correlation with promoter methylation in parental lines and their hybrid. LFC represents expression in log2 fold change and significant *p* values were denoted by * <0.05; ** <0.01; *** <0.001. **b** qRT-PCR analysis of gene expression compared with the RNA-seq data. Relative quantification was obtained through 2^−(ΔΔCT)^ method using GhHIS3 as reference gene

For qRT-PCR validation, 15 DEGs including 8 DEMs were randomly selected. We compared the qRT-PCR results with the RNA-seq data, and the expression patterns were consistent, with a correlation of R^2^ = 0.8205 (Table S11, Fig. 12), demonstrating that the RNA-seq data were credible.

## Discussion

Heterosis means that an offspring surpasses either parent in desired traits. Hybrid breeding has harnessed the power of heterosis to increase the quality and quantity of crops worldwide. Traits contributing to cotton yield exhibit heterosis in both intraspecific and interspecific crosses. Researchers are making great efforts to figure out the complicated genetic basis of crop heterosis. However, much work is still needed, especially with the complicated genome of tetraploid cotton. We generated temporally dynamic transcriptome, small RNAome, and DNA methylome atlases in an elite hybrid and its parents during the three flowering developmental stages, which allowed us to identify differences in coexpression networks between the hybrid and its parents that underpin dynamic regulation of growth heterosis. Our results show that the hybrid and its parents have highly conserved gene regulatory networks with different levels of module gene expression and structural network patterns.

### Dominant role of non-additive expression in establishing cotton heterosis

Variations in gene expression patterns may result in hybrid heterosis. Gene expression patterns can be classified as additive or non-additive based on transcriptome data from hybrids and parents [33]. According to some studies, non-additive expression hampers plant heterosis [34]. For instance, a transcriptomic investigation of mature embryos from the parental lines and an elite super-hybrid rice called LYP9 revealed that additive expression was the major cause of heterosis while non-additive expression was detrimentally related to heterosis [35]. Furthermore, a considerable number of genes display additive expression patterns in genome-wide transcript analysis of maize hybrids and their inbred parents, which are assumed to be the main source of yield heterosis [36]. However among different species with diverse heterotic rates such as tea [37], Soybean [38], cotton [39], and rice [40], when compared to hybrids without heterosis, hybrids with heterosis are more likely to exhibit non-additive expression. A high fraction of genes with non-additive expression patterns were identified as the source of heterosis in pepper [41]. Increase in photosynthetic capacity, cell size, and cell count are caused by non-additively expressed genes in Arabidopsis, suggesting that these genes may be crucial for biomass heterosis [42]. In this study, we detected significant transcriptional reprogramming after hybridization in the G. *hirsutum*, with 10–22% of genes showing non-additive expression. Of all non-additive genes, 69–71% showed dominance of the higher parent (ELD) and 5% showed over-dominance. This outcome is congruent with a study on *B. napus*, in which between 70 and 80% of the non-additive genes in the F1 hybrid had strong parental expression throughout the early stages of flower formation [43]. Analysis of homeolog expression in resynthesized allopolyploid canola showed that ELD is primarily responsible for growth vigour [22]. According to the traditional genetic concept, the genomes of the two parents release their beneficial alleles into the F1 hybrids’ nucleus, which results in dominance of the higher parent and growth vigour of the hybrid [44]. These results support the concept that while numerous molecular processes contribute to heterosis, the dominance of high parental levels may be particularly relevant [45].

Following that, we ran GO and KEGG enrichment analysis for the genes with nonadditive expression. Most of the genes were discovered to be enriched in pathways that are fundamentally associated with heterosis, including signal transduction for plant hormones, defense, and primary metabolism. Among the DEGs related to defense and hormones, found in the hybrid, PRs and DEGs encoding the enzymes involved in SA and auxin production showed significantly different expression patterns than MPV. Notably, most PR genes that were downregulated in the hybrid were methylated. The altered gene activity may be responsible for the development of the heterotic phenotype, since auxin and SA have vital roles in plant defense and in the control of structure and growth [46–48]. The genes involved in SA were significantly downregulated in F1 compared with MPV, with the exception of PAL1 and PAL17. Several studies have found that lower SA levels are associated with greater cell wall expansion and higher green biomass [49, 50]. Notably, reduced expression of basal defense genes in hybrid plants may lead to lower disease resistance. These results suggest that expansion is favored over disease resistance. In the absence of disease infestations, the hybrid may exhibit a weaker basal defense response but may spend more energy on metabolism.

In addition, hybrid plants have better auxin signalling, resulting in faster development. High expression of auxin-related genes has promoted heterosis in crop plants such as Arabidopsis [51, 52], rice [53] and maize [54]. Four genes inducible by auxin, SAUR, IAA1, IAA24, and PIF4, were overexpressed and unmethylated in the hybrid, possibly affecting plant vigour. The gene SAUR was found to be involved in the auxin signal transduction pathway [55]. In line with what we observed, Huang et al. showed that the gene SAUR can alter the auxin distribution and plays a putative regulatory role in hybrid seed size in *Euryale ferox* Salisbury hybrid seeds [56]. In Arabidopsis hybrids, Phytochrome-Interacting Factor (PIF4), which regulates auxin biosynthesis and action, has a vital role in the development of the hybrid vigour phenotype [51]. Pallavi, et al. (2020), stated that gene activity directed toward IAA was frequently associated with a heterotic phenotype with higher leaf cell number, whereas gene activity directed toward SA was negatively associated with heterosis [9]. High auxin levels are associated with increases in cell wall-modifying enzymes that activate molecular pathways that promote plant growth and development [51]. In addition, changes in flavonoid biosynthetic pathway genes negatively affect polar transport of auxin, and a slight reduction in flavonoid biosynthetic pathway genes can promote plant growth [57]. In potato hybrids, genes related to flavonoid biosynthesis were down-regulated, whereas genes involved in auxin signaling were significantly up-regulated [45]. Here the F1 hybrid also showed downregulation and methylation of two genes (FL and F3H) of the flavonoid biosynthesis pathway compared with MPV.

Plant energy metabolism GO terms such as “lipid and carbohydrate metabolic processes,“ “cellular nitrogen compound catabolic processes”, and “photosynthesis,“ were also enhanced, according to the GO enrichment study. F1 exhibited significantly higher expression of DEGs involved in various metabolic pathways than the parental lines. Sugar transporters (SWEET17 and STP1) and nitrogen and sugar metabolism (sucrose synthase (Sus), starch synthase, and cellulose synthase (CESAs)) were increased in the hybrids compared to MPV, indicating an increase in carbohydrate metabolism. This could be due to the better growth performance of the hybrids [58]. In addition, we observed higher expression of energy-related genes, suggesting that the hybrid has a more efficient energy metabolism. Since most of these genes were up-regulated, they were found to be unmethylated. Sucrose synthase and other metabolic genes related to heterosis have previously been found in cotton [59], pigeon pea [9] and rice hybrids [58]. These findings suggest that heterosis induces fundamental alterations in energy production and response to environmental stimuli in F1 hybrid plants.

### miRNAs key regulators in hybrid performance

There is evidence that hybrid plants exhibit different expression patterns for miRNAs involved in development and vigour compared to their parents [41, 60]. Nonadditive expression of miRNAs in Chinese cabbage results in nonadditive expression of their targets, which might impact growth and vigour of hybrid [61]. We observed that many miRNAs, including several miRNAs with well-characterized functions, were expressed in hybrid cells non-additively. A significant percentage of non-additively expressed miRNAs were found to be inhibited in F1 hybrid. This is similar with findings from research on pepper [60], and Chinese cabbage [61] which demonstrate that other regulators or methylation-type changes can affect mRNA target expression. In the hybrid, the expression of miR164, miR169, and miR408 families are primarily reduced, suggesting overexpression of NAC1, an ABA-responsive transcription factor, and CALS6, which enhances auxin signaling, and controls plant growth and stress tolerance, respectively [62]. Growth-regulating factor (GRF) genes regulate plant growth and development [63]. In addition to six GRF genes, ectopic overexpression of miR396 also inhibits the expression of GIF1, which is a GRF-interacting transcription coactivator involved in proliferation of leaf cell [64]. MiR396 targets in cotton are likewise GRF genes, and our investigation found that miR396 was over dominantly suppressed. Because GRFs may control cell division, it is plausible that miR396 suppression, which leads to GRF induction, increases hybrid biomass relative to its parental inbred lines. This finding is consistent with previous findings in B. *rapa* [61] and B. *napus* [43] indicating that the miR396-GRF pathway is preserved and may be involved in heterosis biomass.

The Squamosa-Promoter Binding Protein-Like (SPL) transcription family is targeted by miR156 [65]. SPL transcription factors perform a variety of tasks. SPL transcription factors, according to omics research, not only have metabolic roles but also have an effect on developmental processes through the regulation of other transcription factor families [66, 67]. There has been a lot of research done on SPL transcription factors in plants, specifically in relation to plant development, especially the shift from vegetative to reproductive stages [68]. In the current study, miR156 was over-suppressed, which results in overexpression of SPL transcription factors. OsSPL14 overexpression in rice increased panicle branching and increased grain yield [69]. Interestingly, three G. *hirsutum OsSPL10* homologs in F1 appear to exhibit enhanced expression throughout the squaring stage (Gh-D11G0821, Gh-D12G0947, and Gh-A12G0866). As a result, it is possible that the miR156-SPL pathway, which is not additively controlled, plays a role in the heterosis of biomass and boll number in G. *hirsutum* hybrid.

In addition, there was a noticeable downregulation of the miR164, miR166, miR167, miR169, miR171, and miR390 in F1 compared to the parental lines. MiRNAs 164, 166, 167, and 390 targeted ARF genes and were controlled by IAA, signifying that they are also associated with the IAA response pathways [70]. Nuclear factor Y (NF-Y), also known as Heme Activator Protein or CCAAT binding factor (CBF), is a miR169 target found in plants, animals, and other eukaryotes (HAP) [71]. The NF-Y complex is comprised of three subunits: NF-YA (CBF-B/HAP2), NF-YB (CBF-A/HAP3), and NF-YC (CBF-C/HAP5), all three of which are vital for CCAAT box binding [72]. Numerous studies in Arabidopsis and other plant species have elucidated the biological functions of each NF-Y subunit and demonstrated that this complex is involved in embryogenesis, gametogenesis, flowering time regulation, seed development, drought tolerance, abscisic acid signalling, endoplasmic reticulum stress response, and primary root elongation [73, 74]. According to these studies, NF-Y genes play a vital part in many aspects of plant life and are important for their survival. Most miR169 family members were significantly repressed in this study, possibly affecting NFY gene expression and thus ABA-dependent transcription, leading to increased internode growth potential in the hybrid. Another family, miR171, targets transcription factors such as SCL6, which control the development of the plant and have links to such biological processes as cell proliferation, differentiation, and branching [75]. Overall, these data indicated that F1 hybrids’ growth vigour and adaptability are most likely controlled by non-additively expressed miRNAs.

### siRNAs act as genetic buffers by suppressing TEs activity and affecting gene expression in the F1 hybrid by manipulating methylation levels

The importance of siRNA-mediated epigenetic changes in the genomic stability of interspecific hybrids and allopolyploids has been demonstrated [76]. Depending on the differences in the 24-nt siRNA clusters presence at certain loci, hybrids might have hyper- (TCMDMRs) or hypo-methylation (TCMDMRs) [77]. In our study, the methylation sites were usually associated with the clusters of 24-nt siRNA in hybrid. About 38% of the methylated loci showed a significant density of 24-ntsiRNA clusters, demonstrating their importance in DNA methylation [78]. In this context, we studied the possible connection between the level of methylation of DNA and expression of 24-nt siRNA clusters in F1 compared with its parents. TCM-DMRs and TCdM-DMRs were found to have a strong correlation with clusters of 24-nt siRNA. TCdM-DMRs had higher 24-nt siRNA expression than TCM genomic regions. TCdM-DMRs are associated with a decrease in CHH methylation, which is thereafter followed by CG and CHG methylation. The variations in methylation loss between contexts of cytosine methylation are most likely due to differences in the mechanisms that keep each context alive [79].

The increased expression of 24-nt-siRNAs identified in the G. *hirsutum* hybrid is comparable to what has been observed in other plant species such as *Brassica napus, Oryza sativa, Arabidopsis thaliana*, and *Cajanus cajan*, where an increase in 24-nt-siRNAs has been observed after hybridization [9, 43, 80, 81]. Additionally, the majority of the *G. hirsutum* hybrid’s increased methylation came from unmethylated cytosines in both parents, suggesting de novo DNA methylation and trans chromosomal methylation activity (TCM) [82]. We observed that the intergenic area, as opposed to the genic region (including 2 kb upstream/downstream of genes and gene-encoding regions), typically contains up to 70% of the methylated cytosines produced by de novo DNA methylation or TCM processes, implying its regulatory role in heterosis [83]. Indeed, the level of methylation in the coding region of a gene is not necessarily directly related to its expression level. A study of *Brassica napus* found that methylation of three quarters of the genes occurred in their immediate vicinity, which includes 1 kb downstream and upstream of the annotated gene [43]. Moreover, Pallavi, et al. (2020) also observed that promoter methylation and methylation of transcriptional regions of protein-coding genes, may have a vital function in gene activity regulation [9]. We also discovered DNA methylation in the promoter and other regulatory transcription regions of protein-coding genes, signifying that it may be important for regulating gene activity [84]. Moreover, the majority of the methylation was observed in the repetitive regions of the DNA and in transposable elements. By limiting their activity, a steady repressive epigenetic process in these regions promotes stability of the genome [85]. These findings imply that clusters of 24-nt siRNA are required for higher methylation of DNA in hybrids, and the TCM/TCdM-DMRs context pattern suggests that 24-nt siRNA clusters are associated with the lower activity of TE in hybrids.

## Conclusions

Heterosis occurs as a result of genome interactions and leads to complex variation at genetic, epigenetic, and metabolic levels [86]. It is generally ineffective to assess complex biological traits using only a single omics technique. This study, the hybrid with superior yield was screened through multi-location field trials during different years. Phenotypic evaluation suggested that this hybrid yielded on average 16% more seed cotton than its mean parent. Several -omics experiments were carried out on this hybrid and its two inbred parent lines, using different samples collected throughout the squaring stage to acquire comprehensive evidence on the function of crucial changes in gene expression and epigenetic mechanisms which lead to heterosis. Comparative transcriptomic analysis revealed significant variations in gene expression (MPV-DEGs) in the hybrid and its parents. These genes were primarily associated with hormone transduction, metabolic pathways, response to stress, ATP and protein binding, cell wall, sugar metabolism, and signal transduction of plant hormones. Analysis of the dominance of expression levels revealed that most of the DEGs of the hybrid were biased toward the high parental expressions. The amount of 24-nt small RNA (siRNA) in the hybrid was higher than in the parents at the loci where there was a discernible difference in the amount of 24-nt siRNA between the parents. Most of these regions with elevated levels of 24-nt sRNA were related with transposon elements, genes, and the regions flanking them, which may impact gene expression. Moreover, epigenetic changes were also observed in DEGs involved in heterosis-related regulatory pathways. In the hybrid, we also discovered a number of common and unique miRNAs that are crucial regulators of target genes related to plant development, defense, and stress tolerance. In addition, we observed that 24-nt-sRNA levels correlated with variation in DNA methylation of TEs and expression of protein-coding genes. We suggest that such epigenetically driven changes in gene activity may lead to hybrid vigour and that epigenetic variation between hybrids and parents may lead to increased variability in allele expression, which may contribute to heterosis. Taken together, we propose that hormones, plant growth regulators, plant-pathogen interactions and stress responses controlled by both miRNAs and DNA methylation due to variation in 24-nt siRNAs are the primary mechanisms by which ELD and non-additively expressed genes contribute to heterosis. Thus, our results shed light on the key regulatory networks and genes associated with hybrid vigour in allotetraploid cotton.

## Materials and methods

### Plant material, growth conditions and trait investigation

In 2018, our research team used 10 inbred parent lines to produce 21 intraspecific F1 upland cotton hybrids. A trial was conducted for two years to measure the degree of heterosis in yield traits between the hybrids and their parents, with seed sown in mid-April of each year for two years. Number of bolls per plant (BN), boll weight (BW**)**, seed cotton yield **(**SCY), lint yield **(**LY**)**, and lint percentage (LP); number of reproductive branches, and number of fruit units were measured. Mean parental value (MPV) was calculated as follows: MPV = ((F1-MP)/ MP)100%, and better parental heterosis (BPH) was calculated as follows: BPH = (F1-BP)/ BP, wherein F1 indicates the trait performance value for each trait in the F1 offspring, MP indicates the mean value of this trait for the parents, and BP indicates the value of this trait for the better parent [87]. The t-test was used to evaluate the hypotheses. On the basis of the degree of phenotypic heterosis in yield parameters, a high hybrid and its inbred line parents were selected for transcriptome, small RNAome and methylome studies at the squaring stage, the beginning of the reproductive phase in cotton. Cotton seeds were collected from Cotton Research Institute of Iran (Gorgan, Iran) and planted at Hashem Abad research station (Gorgan, Iran) under controlled climatic conditions of 70% relative humidity and 20/15 C (14 h/10 h) day/night temperature regime. Seed collection and the whole experiment were carried out according to the national guidelines [88]. The inbred lines utilized in this study underwent six generations of self-fertilization to ensure their purity.

### Whole-genome DNA resequencing, mRNA, sRNA, bisulfate sequencing, and bioinformatics analysis

Methods S1 provides detailed information on plant material, growth conditions and trait assays, nucleic acid extraction, whole genome DNA resequencing, mRNA (RNA sequencing) and small RNA (miRNA and siRNA) sequencing, bisulfate sequencing (DNA methylation sequencing), bioinformatics analyses and quantitative RT-qPCR expression validation of DEGs and DMGs (Fig. S1).

### Supplementary Information

**Additional file 1: Table S1.** Mean of mid and better parent heterosis observed for yield traits in two location and two-year field experimentation. **Table S2.** Statistics of RNA sequencing data production. **Table S3.** Overlapping differentially expressed genes among the in MH, SM, and 1DPA in hybrid. **Table S4.** Statistics of small RNA sequencing data production. **Table S5. ** Number of the non-additively expressed genes had siRNA clusters in coding regions or 2kb flanking regions in MH, SM, and 1DPA. **Table S6a.** List of known differentially expressed miRNA in F1 and its Parents. **S6b.** List of target prediction of known miRNAs. **S6c**. List of target prediction of novel miRNAs. **Table S7.** Alignment summary statistics of whole-genome bisulfite sequencing (WGBS). **Table S8.** List of common DMRs among parental lines and hybrids. **Table S9.** Association of DMRs and sRNA among hybrid and its parental lines. **Table S10.** REVIGO combines the biological process GO with hybrid MPV DEGs. The unified terms of REVIGO GO are organized by major groups. To make understanding the data even easier, clusters are grouped into "main clusters" with broad categories. **Table S11.** Details of the primers used to validate RNA-seq and bisulfite sequencing data.

**Additional file 2: Figure S1.** The overall workflow was carried out to study the heterosis mechanism in cotton. Hybrid and its parental lines were selected for this analysis. First, the parental lines and hybrid were phenotypically characterized at 20, 40, and 60 days after sowing. The 40-day seedlings were used to prepare libraries RNA sequencing, small RNA, and DNA methylation. Several bioinformatics pipelines were used to decipher the genome-wide data and answer the biological questions. As mentioned in the figure, we performed several different analyses on the datasets to understand heterosis in allotetraploid cotton. **Figure S2.** The mapped region’s statistics of all 27 sequenced libraries of parents and hybrid. Here, MH: match-head, SM: square growth midpoint, 1DPA: a day post anthesis ovule, (a) paternal line, and (b) F1, and (c) represents Maternal line respectively. **Figure S3. **Principal component analysis for all samples. In the figure, MH: match-head, SM: square growth midpoint, 1DPA: a day post anthesis ovule, maternal parent, paternal parent and F1 are represented in different colour (three biological replicates). **Figure S4. **Total number of expressed genes for each sample. In this figure, MH: match-head, SM: square growth midpoint, 1DPA: a day post anthesis ovule, maternal parent, paternal parent and F1 are represented in different colour (three biological replicates). **Figure S5. **(a) Breakdown of DEGs in the F1 hybrid showing the number of differentially expressed genes that were unique in one sample or overlapping in two or three samples used in this study. (b) Overlapping genes between MPV-DEGs and M-F DEGs in all three developmental stages.** Figure S6. **Prominent GO terms enriched in the genes with a trans effect. (a) Gene Ontology (GO) and (b) KEGG enrichment analyses for all trans effects in genes in all datasets (www.kegg.jp/kegg/kegg1.html, For previous uses, the Kanehisa laboratory have happily provided permission). **Figure S7.** (a). Class distribution analysis of filtered reads in hybrid and parental lines showed that 21 and 24 nucleotide classes were the most abundant groups. (b) Distribution of the 24-nucleotide siRNA clusters in the 2 kb upstream, 2 kb downstream, transcribed region and transposable elements in the three samples. (c) Length distribution of the 24-nucleotide siRNA clusters in the three samples. **Figure S8.** (a) Principal component analysis for all samples (two replicates). (b) Pearson correlation coefficients between different sample replicate used for whole-genome bisulfite sequencing (WGBS) analysis. **Figure S9.** Average methylation distribution across different genomic regions. Metaplots representing methylation distribution at genomic elements and their flanking regions (a–d) transposable-elements (TEs) body including DNA-elements, LTRs, non-LTRs and gene-body, respectively, along with their 1.5 kb flanking regions. Methylation at gene/TE -body and flanking regions was analyzed across 20 and 30 bins, respectively. (e, f) exon-body and intron body methylation along with their 1.5 kb flanking regions.** Figure S10.** Boxplots showing the methylation levels of CG, CHG, and CHH on Rich-siRNA clusters and Poor-siRNA clusters in hybrid and its parent.

**Additional file 3. **Methods S1.

## Data Availability

All data supporting the findings of this study are available from the corresponding author upon reasonable request. All the raw sequences for the samples were compiled at https://www.ncbi.nlm.nih.gov/bioproject/PRJNA973929 with the SRA ID SRR24677352, SRR24676578, SRR24676581, SRR24676223, SRR24675764, SRR24676220, SRR24676178, SRR24676002, SRR24675763, and SRR24675543.

## Abbreviations

SA: Salicylic acid
ARF: Auxin response factor
cDNA: Complementary DNA
DEMs: Differentially expressed miRNAs
FDR: False Discovery Rate
GO: Gene Ontology
KEGG: Kyoto Encyclopedia of Genes and Genomes
MiRNA-seq: Micro-RNA sequencing
miRs: miRNAs
NCBI: National Center for Biotechnology Information
GBM: Gene Body Methylation
MH: Match-Head
SM: Square Growth Midpoint
1DPA: One-Day Post Anthesis
DEGs: Differentially expressed genes
ELD: Expression Level Dominance
SNPs: Single Nucleotide Polymorphisms
TEs: Transposon Elements
TCM: Transchromosomal Methylation
TCdM: Transchromosomal Demethylation
DMRs: Differentially Methylated Regions
RdDM: RNA-directed DNA methylation
