# Supplementary material for: Dynamic roles of small RNAs and DNA methylation associated with heterosis in allotetraploid cotton (Gossypium hirsutum L.)
Source: BMC Plant Biol. 2023 Oct 13;23:488. doi: 10.1186/s12870-023-04495-2 (PMC10571366; doi:10.1186/s12870-023-04495-2)
Supplement: Supplementary file 2 — Additional file 2: Figure S1. The overall workflow was carried out to study the heterosis mechanism in cotton. Hybrid and its parental lines were selected for this analysis. First, the parental lines and hybrid were phenotypically characterized at 20, 40, and 60 days after sowing. The 40-day seedlings were used to prepare libraries RNA sequencing, small RNA, and DNA methylation. Several bioinformatics pipelines were used to decipher the genome-wide data and answer the biological questions. As mentioned in the figure, we performed several different analyses on the datasets to understand heterosis in allotetraploid cotton. Figure S2. The mapped region’s statistics of all 27 sequenced libraries of parents and hybrid. Here, MH: match-head, SM: square growth midpoint, 1DPA: a day post anthesis ovule, (a) paternal line, and (b) F1, and (c) represents Maternal line respectively. Figure S3. Principal component analysis for all samples. In the figure, MH: match-head, SM: square growth midpoint, 1DPA: a day post anthesis ovule, maternal parent, paternal parent and F1 are represented in different colour (three biological replicates). Figure S4. Total number of expressed genes for each sample. In this figure, MH: match-head, SM: square growth midpoint, 1DPA: a day post anthesis ovule, maternal parent, paternal parent and F1 are represented in different colour (three biological replicates). Figure S5. (a) Breakdown of DEGs in the F1 hybrid showing the number of differentially expressed genes that were unique in one sample or overlapping in two or three samples used in this study. (b) Overlapping genes between MPV-DEGs and M-F DEGs in all three developmental stages. Figure S6. Prominent GO terms enriched in the genes with a trans effect. (a) Gene Ontology (GO) and (b) KEGG enrichment analyses for all trans effects in genes in all datasets (www.kegg.jp/kegg/kegg1.html, For previous uses, the Kanehisa laboratory have happily provided permission). Figure S7. (a). Class distribution [file 12870_2023_4495_MOESM2_ESM.docx]

**Methods S1**

**RNA extraction, Transcriptome sequencing and data analysis**

For RNA samples, flower buds were collected at three developmental stages "match-head" (MH), "square growth midpoint" (SM), and one-day post anthesis (1DPA) ovule in the three biological replicates for all the three materials from field trial conducted in 2021. The isolation of total RNA was done using a hot borate RNA isolation method, from cotton seedlings [[1](#_ENREF_1)]. Paired-end sequencing (150 bp) was executed on the Illumina Hiseq 2500 platform in accordance with the suggested procedure after initial quality assessments and preparations. FastQC [[2](#_ENREF_2)] and PRINSEQ [[3](#_ENREF_3)] were used for quality control. TopHat2 [[4](#_ENREF_4)] was used to align the clean reads to the upland cotton genome. The assembly of the mapped reads was done using Cufflinks. After the final transcriptome generation, StringTie 1.3 was used with Ballgown [[5](#_ENREF_5)] to measure the levels of expression. Using String-Tie 1.3, mRNAs expression profiles were calculated in the FPKM form. For the identification of the differentially expressed mRNAs between the two samples, the criteria |log2FC| ≥ 2 and P-adj (adjusted P-value)≤0.05 were used [[6](#_ENREF_6)]. The Density Estimator of the R software package was used to carry out expression-level dominance analysis according to the method described by [Shahzad, Zhang, Guo, Qi, Bao, Zhang, Zhang, Wang, Tang and Qiao [7]](#_ENREF_7) and using this method, the expression statistics between the two parents and their resultant hybrid were divided into 12 possible groups. Expression of genes in hybrids can be additive (first and second groups), paternal expression dominant (third and fourth groups), maternal expression dominant (fifth and sixth groups), and transgressive expression lower or higher than both parents (seventh to twelfth groups). To compensate for gene length bias in DEGs, GO developed an enrichment analysis of differentially expressed genes (DEGs) based on the decentralized hypergeometric Wallenius distribution. Statistical enrichment of DEGs in KEGG pathways ([www.kegg.jp/kegg/kegg1.html](http://www.kegg.jp/kegg/kegg1.html)) was investigated using the KOBAS tool. Clustering of highly enriched functional GO terms was performed using the REViGO tool (Function).

**Small RNA sequencing and data processing**

Polyacrylamide gel electrophoresis was used to separate sRNAs from total RNAs. The 15–30 nt-long RNAs were extracted and eluted using 0.3M NaCl, from the gel. After being precipitated in ethanol, small RNAs were dissolved in 5μl of RNase-free water. Following the instructions provided by the manufacturer, NEBNext1 Multiplex Small RNA Library Prep Set was used to create small RNA-seq libraries with three biological replicates (NEB, Ipswich, Massachusetts). The sRNA-seq libraries were sequenced using the Illumina platform. The first 30 nucleotide adaptor sequences were then deleted using a custom Perl script. To avoid including datasets with tRNA products and potentially degraded rRNA, the trimmed sRNA reads were compared to the tRNA and rRNA databases of NCBI. Using Bowtie, [[8](#_ENREF_8)], the remaining trimmed reads were mapped to the cotton reference genome, with only perfect matches allowed. Only the reads that were mapped to unique loci, after mapping, were taken into consideration for further analysis. A cluster with at least three small RNA reads was designated as a sRNA cluster, and sRNA clusters separated by 200 nt were combined.

Moreover, to identify the conserved miRNAs, the sequences, were aligned with known miRNAs from miRBase [http://microrna.sanger.ac.uk/seguence/index.hMHl](http://microrna.sanger.ac.uk/seguence/index.html) (Release 22.1) with no more than two mismatches, where gaps are counted as mismatches. The flanking genomic sequence of certain small RNAs were folded with MIREAP <https://sourceforge.net/projects/mireap/>, followed by secondary structure prediction with Mfold, which yielded potential novel miRNAs [[9](#_ENREF_9)]. The miRNA precursor sequences that can fold into and produce a hairpin secondary structure including the 21 nt mature miRNA sequence from one arm and the miRNA* generated from the opposite arm, both forming a duplex with two nucleotide 3' overhangs, were key criterion for choosing miRNA candidates [[10](#_ENREF_10)]. The approach and criteria for miRNA target prediction were followed as described by [Ghorbanzadeh, Hamid, Jacob, Mirzaei, Zeinalabedini, Abdirad, Atwells, Haynes, Ghaffari and Salekdeh [11]](#_ENREF_11), [Guo, Kuang, Zhao, Deng, He, Wan, Tao, Wang, Wei and Li [12]](#_ENREF_12) and [Liu, Liu, Zhang, Liang, Luan and Ma [13]](#_ENREF_13). In this study, only three mismatches between potential mRNA targets and miRNA sequences were permitted. The predicted targets' biological functions were obtained from the Universal Protein Resource (<http://www.uniprot.org>).

**DNA isolation, whole genome bisulfite sequencing, alignment and identification of differentially methylated regions (DMRs)**

The cotton samples used for bisulfite sequencing were the same as those used for transcriptome analysis, and there were two biological replicates. The CTAB method with slight modifications was used to isolate genomic DNA from the cotton samples [[14](#_ENREF_14)]. To prepare the library for bisulfite sequencing, high-quality DNA from all samples was sonicated to an average size of 100 to 300 bp (Covaris, Massachusetts, USA). After end-repairing genomic segments, the ends were ligated with TrueSeq methylated adaptors. Sodium bisulfite was used to treat the genome fragments ligated with the adaptors [[15](#_ENREF_15)]. Desalting, size selection, PCR amplification, and size selection were carried out following bisulfite treatment. Deep bisulfite sequencing (> 30) on the HiSeq 2000 platform was then performed on the relevant libraries (Illumina, San Diego, USA). To eliminate low-quality reads and adaptor sequences, raw reads were cleaned up with the NGSQC Toolkit (v2.3) using default parameters [[16](#_ENREF_16)]. Duplicate reads were removed by mapping to the cotton genome (MH-1 genome sequence) with Bismark (v0.8) using default parameters [[17](#_ENREF_17)].

When the high-quality filtered reads were aligned to the cotton chloroplast genome to assess the bisulfite conversion efficiency, it was found that more than 99% of the cytosines in the genome were changed to thymine(s) in the chloroplast, indicating a very high bisulfite conversion. The methylated cytosines (mCs) in the cotton genome were detected with a significance value of 0.001 and a sequencing depth of 5 reads, as described in [Zhang, Guo, Qi, Zhang, Tang, Wang, Qiao, Zhang, Feng and Zuo [18]](#_ENREF_18). The degree of methylation was calculated by counting the number of methylation call at a particular cytosine site out of all reads in the sequencing data at that site [[18](#_ENREF_18)]. Circos plots with a window size of 100 kb were used to visualize DNA methylation patterns in the cotton genome. Modified Perl scripts were used to assess the DNA methylation density of genes/TEs and their 2 kb surrounding regions.

Differential methylation analysis between hybrid and its parental lines was performed for each genotype within each 100-bp bin in the cotton genome. Differential methylation analysis was performed for bins containing at least three cytosine residues and covered by at least five reads. The methylation levels of the hybrid and its parental lines were compared in two bins with the same genomic coordinates. The Fisher's exact test followed by a sliding linear model (SLIM) correction was used to determine the differentially methylated bins with a difference of at least 20% in the methylation level with a q-value of ≤0.01, as described in [[19](#_ENREF_19)]. The consecutive differentially methylated bins (within a radius of 50 bp) were pooled and their distribution in different sequence contexts within the gene body and the 2 kb surrounding regions were analyzed, to identify the DMRs. Sites were classified into methylation patterns in the hybrid by comparing the level of methylation in F1 with the mean values of the parents (MPV), also using edgeR. A paired t-test with FDR and p-value of 0.01 was used to perform a significance test.

**Quantitative RT-qPCR expression validation of DEGs and DMGs**

Fifteen DEGs, including eight differentially methylated genes (DMGs), were selected for qRT-PCR validation, and Oligo 7 primer analysis software [[20](#_ENREF_20)], was used to design gene-specific qRT-PCR primers, which were subsequently synthesized commercially (BioSune Biotechnology, Shanghai, China). For qRT-PCR, 1 ng of total RNA (the same samples used in WGBS and RNAseq) was initially used for synthesizing first-strand cDNA with a PrimeScript. The RT Perfect Real Time Reagent Kit (RR037A, Takara, Japan) was used as directed by the manufacturer. The qRT-PCR was then carried out on a Mastercycler® ep realplex using TransStart® Top Green qPCR SuperMix (AQ131, TransGen Biotech, Beijing, China). Using cotton HISTONE H3 as an internal control and using the 2-^ΔΔCt^ method, the relative expression level was evaluated [[21](#_ENREF_21)].

[1] R. Hamid, H. Marashi, R.S. Tomar, S. Malekzadeh Shafaroudi, P.H. Sabara, Transcriptome analysis identified aberrant gene expression in pollen developmental pathways leading to CGMS in cotton (*Gossypium* *hirsutum* L.), PloS one 14(6) (2019) e0218381.

[2] C.-C. Lo, P.S. Chain, Rapid evaluation and quality control of next generation sequencing data with FaQCs, BMC bioinformatics 15(1) (2014) 1-8.

[3] V.A. Cantu, J. Sadural, R. Edwards, PRINSEQ++, a multi-threaded tool for fast and efficient quality control and preprocessing of sequencing datasets, PeerJ Preprints 7 (2019) e27553v1.

[4] D. Kim, G. Pertea, C. Trapnell, H. Pimentel, R. Kelley, S.L. Salzberg, TopHat2: accurate alignment of transcriptomes in the presence of insertions, deletions and gene fusions, Genome biology 14(4) (2013) 1-13.

[5] M. Pertea, D. Kim, G.M. Pertea, J.T. Leek, S.L. Salzberg, Transcript-level expression analysis of RNA-seq experiments with HISAT, StringTie and Ballgown, Nature protocols 11(9) (2016) 1650-1667.

[6] F.J. Thoppurathu, Z. Ghorbanzadeh, A.K. Vala, R. Hamid, M. Joshi, Unravelling the treasure trove of drought-responsive genes in wild-type peanut through transcriptomics and physiological analyses of root, Functional & Integrative Genomics 22(2) (2022) 215-233.

[7] K. Shahzad, X. Zhang, L. Guo, T. Qi, L. Bao, M. Zhang, B. Zhang, H. Wang, H. Tang, X. Qiao, Comparative transcriptome analysis between inbred and hybrids reveals molecular insights into yield heterosis of upland cotton, BMC plant biology 20(1) (2020) 1-18.

[8] W.B. Langdon, Performance of genetic programming optimised Bowtie2 on genome comparison and analytic testing (GCAT) benchmarks, BioData mining 8(1) (2015) 1-7.

[9] M. Zuker, Mfold web server for nucleic acid folding and hybridization prediction, Nucleic acids research 31(13) (2003) 3406-3415.

[10] B.C. Meyers, M.J. Axtell, B. Bartel, D.P. Bartel, D. Baulcombe, J.L. Bowman, X. Cao, J.C. Carrington, X. Chen, P.J. Green, Criteria for annotation of plant MicroRNAs, The Plant Cell 20(12) (2008) 3186-3190.

[11] Z. Ghorbanzadeh, R. Hamid, F. Jacob, M. Mirzaei, M. Zeinalabedini, S. Abdirad, B.J. Atwells, P.A. Haynes, M.R. Ghaffari, G.H. Salekdeh, MicroRNA Profiling of Root Meristematic Zone in Contrasting Genotypes Reveals Novel Insight into in Rice Response to Water Deficiency, Journal of Plant Growth Regulation (2022) 1-21.

[12] Z. Guo, Z. Kuang, Y. Zhao, Y. Deng, H. He, M. Wan, Y. Tao, D. Wang, J. Wei, L. Li, PmiREN2. 0: from data annotation to functional exploration of plant microRNAs, Nucleic acids research 50(D1) (2022) D1475-D1482.

[13] J. Liu, X. Liu, S. Zhang, S. Liang, W. Luan, X. Ma, TarDB: an online database for plant miRNA targets and miRNA-triggered phased siRNAs, BMC genomics 22(1) (2021) 1-12.

[14] V. Rathod, R. Hamid, R.S. Tomar, S. Padhiyar, J. Kheni, P. Thirumalaisamy, N.S. Munshi, Peanut (*Arachis* *hypogaea*) transcriptome revealed the molecular interactions of the defense mechanism in response to early leaf spot fungi (*Cercospora* *arachidicola*), Plant Gene 23 (2020) 100243.

[15] M.S. Rajkumar, R. Shankar, R. Garg, M. Jain, Bisulphite sequencing reveals dynamic DNA methylation under desiccation and salinity stresses in rice cultivars, Genomics 112(5) (2020) 3537-3548.

[16] M.S. Rajkumar, K. Gupta, N.K. Khemka, R. Garg, M. Jain, DNA methylation reprogramming during seed development and its functional relevance in seed size/weight determination in chickpea, Communications biology 3(1) (2020) 1-13.

[17] Q. Zhao, J. Wu, G. Cai, Q. Yang, M. Shahid, C. Fan, C. Zhang, Y. Zhou, A novel quantitative trait locus on chromosome A9 controlling oleic acid content in Brassica napus, Plant biotechnology journal 17(12) (2019) 2313-2324.

[18] M. Zhang, L. Guo, T. Qi, X. Zhang, H. Tang, H. Wang, X. Qiao, B. Zhang, J. Feng, Z. Zuo, Integrated methylome and transcriptome analysis between the CMS-D2 Line ZBA and its maintainer line ZB in upland cotton, International journal of molecular sciences 20(23) (2019) 6070.

[19] S. Kumar, K. Seem, S. Kumar, K. Vinod, V. Chinnusamy, T. Mohapatra, Pup1 QTL Regulates Gene Expression Through Epigenetic Modification of DNA Under Phosphate Starvation Stress in Rice, Frontiers in plant science 13 (2022).

[20] W. Rychlik, OLIGO 7 primer analysis software, PCR primer design (2007) 35-59.

[21] M. Imran, S. Shafiq, M.A. Farooq, M.K. Naeem, E. Widemann, A. Bakhsh, K.B. Jensen, R.R.-C. Wang, Comparative genome-wide analysis and expression profiling of histone acetyltransferase (HAT) gene family in response to hormonal applications, metal and abiotic stresses in cotton, International journal of molecular sciences 20(21) (2019) 5311.
