# Supplementary material for: Dynamic roles of small RNAs and DNA methylation associated with heterosis in allotetraploid cotton (Gossypium hirsutum L.)
Source: BMC Plant Biol. 2023 Oct 13;23:488. doi: 10.1186/s12870-023-04495-2 (PMC10571366; doi:10.1186/s12870-023-04495-2)
Supplement: Supplementary file 3 — Additional file 3. Methods S1. [file 12870_2023_4495_MOESM3_ESM.docx]

**Supplementary Figures**

**Figure S1.**


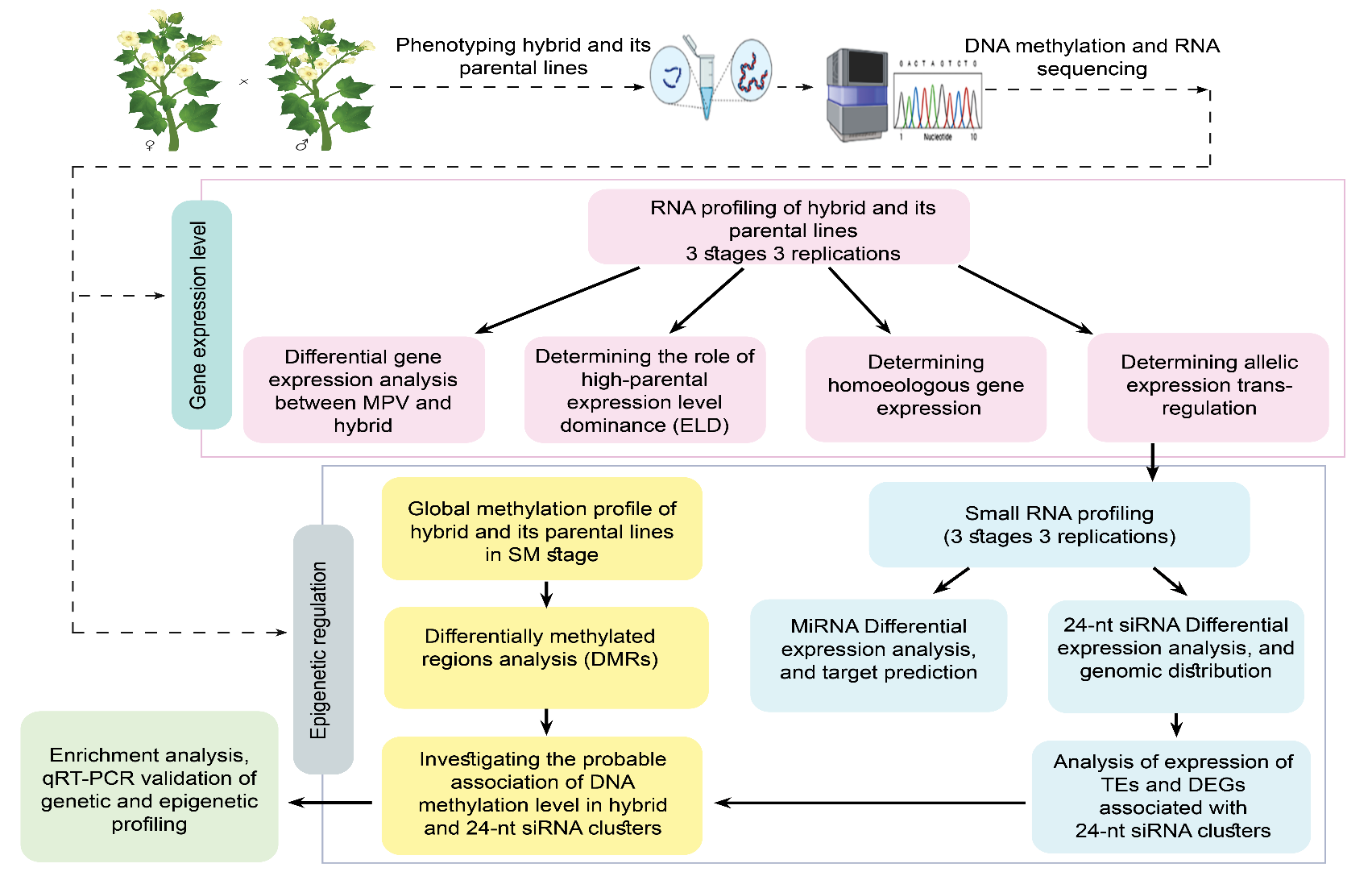


**Figure S1.** The overall workflow was carried out to study the heterosis mechanism in cotton. Hybrid and its parental lines were selected for this analysis. First, the parental lines and hybrid were phenotypically characterized at 20, 40, and 60 days after sowing. The 40-day seedlings were used to prepare libraries RNA sequencing, small RNA, and DNA methylation. Several bioinformatics pipelines were used to decipher the genome-wide data and answer the biological questions. As mentioned in the figure, we performed several different analyses on the datasets to understand heterosis in allotetraploid cotton.

**Figure S2.**


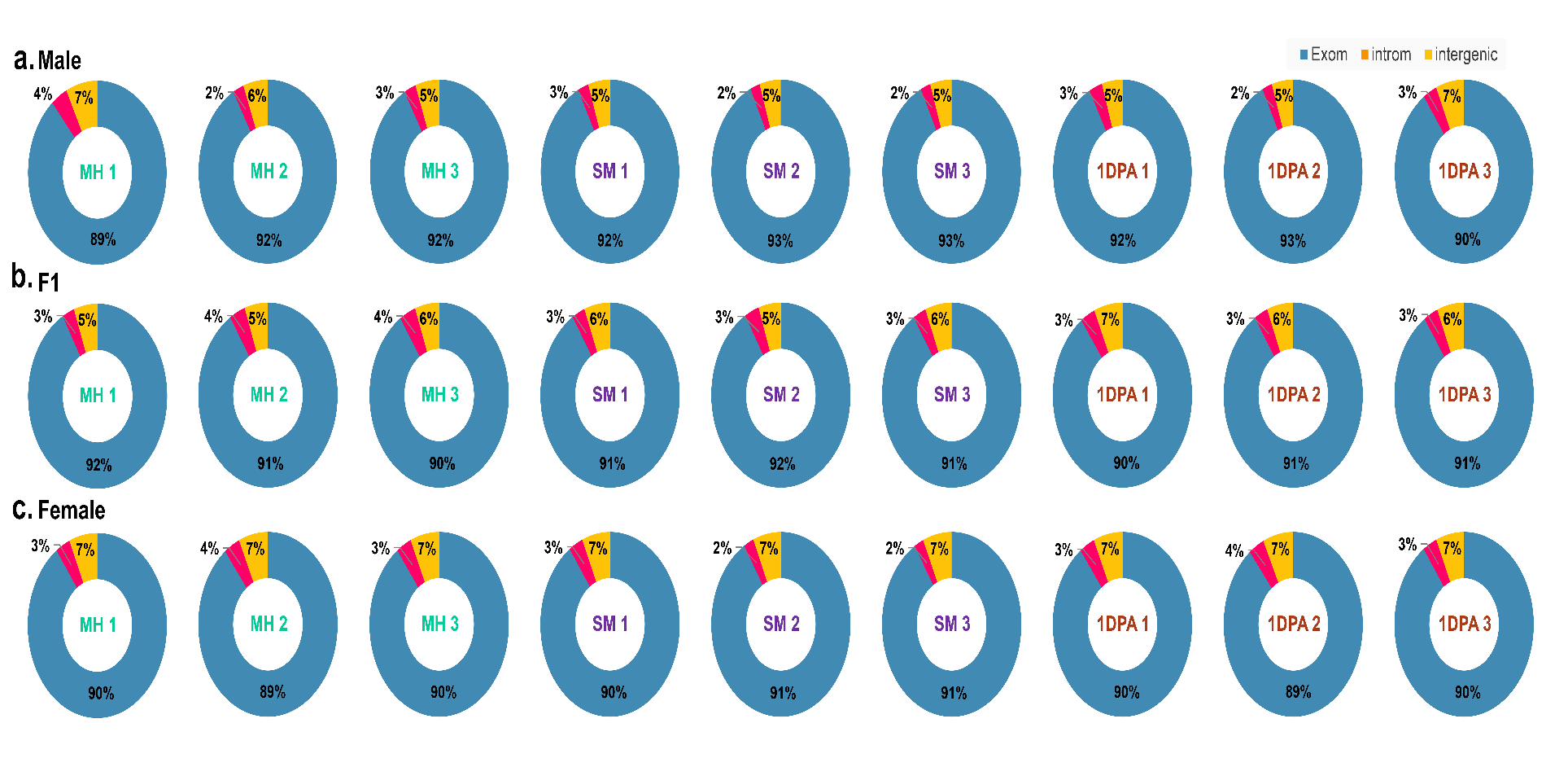


[**Figure S2.**](https://static-content.springer.com/esm/art%3A10.1186%2Fs12870-020-02442-z/MediaObjects/12870_2020_2442_MOESM1_ESM.png) The mapped region’s statistics of all 27 sequenced libraries of parents and hybrid. Here, MH: match-head, SM: square growth midpoint, 1DPA: a day post anthesis ovule, (a) paternal line, and (b) F1, and (c) represents Maternal line respectively.

[**Figure S3.**](https://static-content.springer.com/esm/art%3A10.1186%2Fs12870-020-02442-z/MediaObjects/12870_2020_2442_MOESM1_ESM.png)


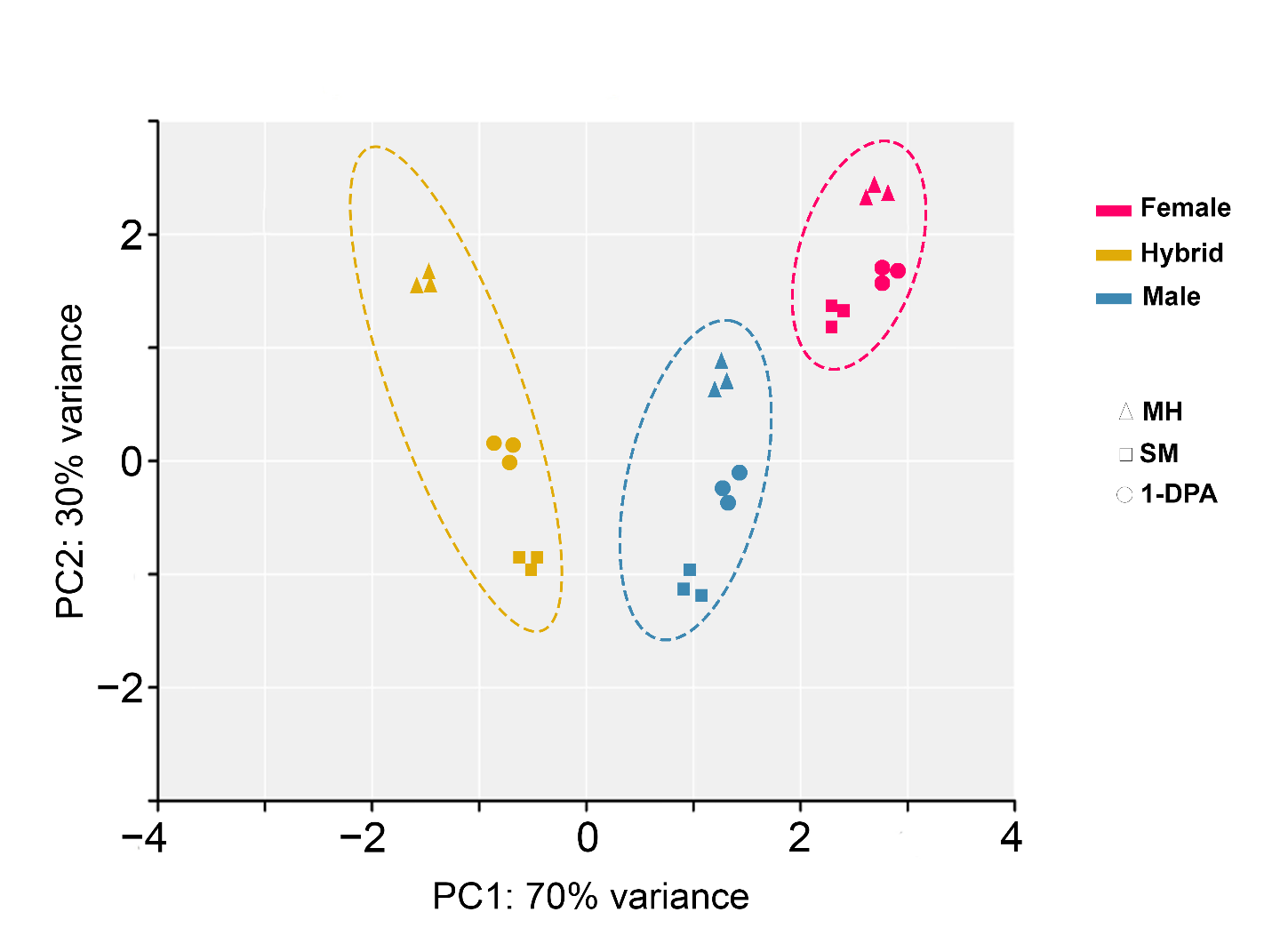


**[Figure S3.](https://static-content.springer.com/esm/art%3A10.1186%2Fs12870-020-02442-z/MediaObjects/12870_2020_2442_MOESM1_ESM.png)** Principal component analysis for all samples. In the figure, MH: match-head, SM: square growth midpoint, 1DPA: a day post anthesis ovule, maternal parent, paternal parent and F1 are represented in different colour (three biological replicates).

[**Figure S4.**](https://static-content.springer.com/esm/art%3A10.1186%2Fs12870-020-02442-z/MediaObjects/12870_2020_2442_MOESM1_ESM.png)


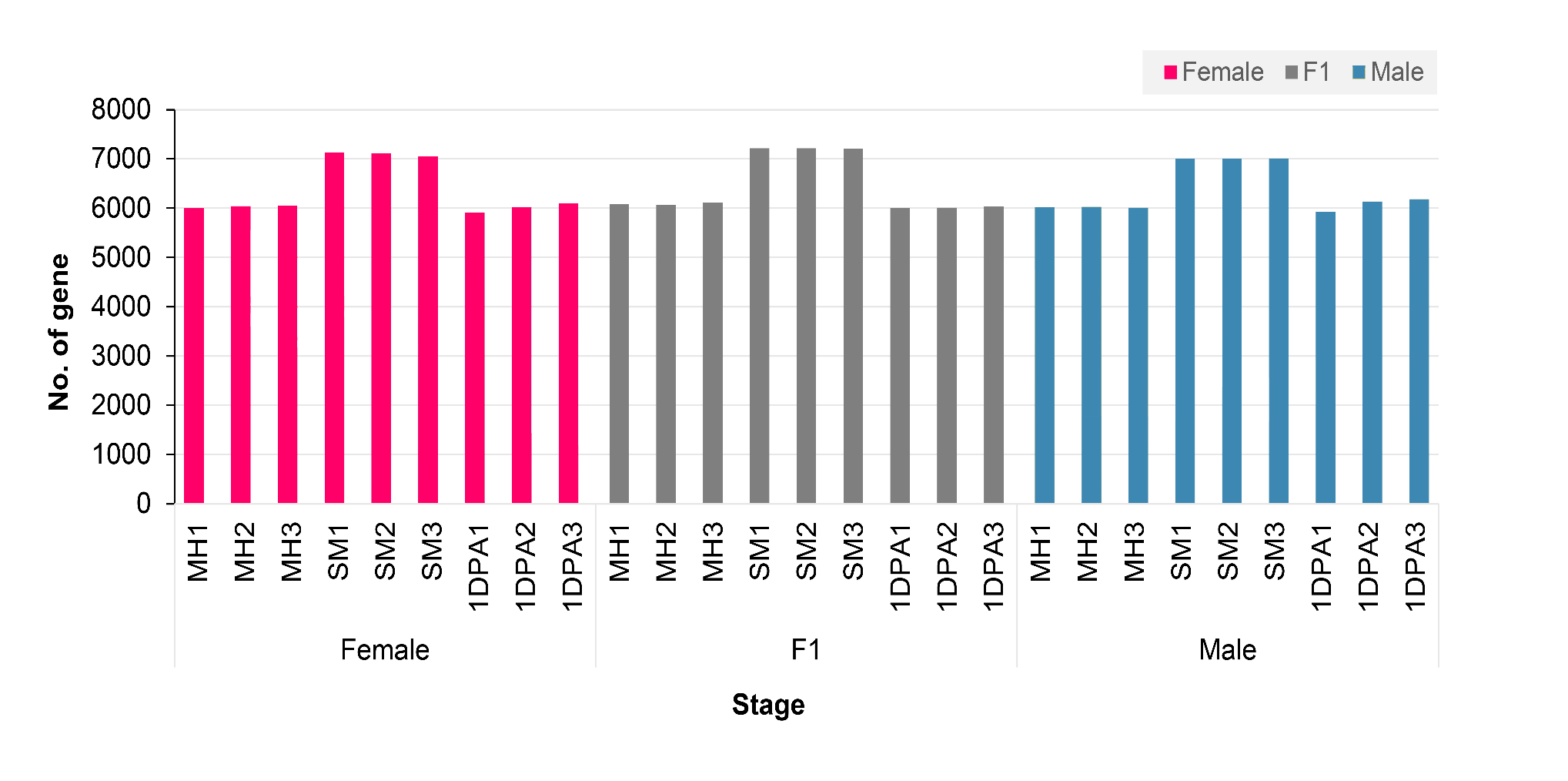


[**Figure S4.**](https://static-content.springer.com/esm/art%3A10.1186%2Fs12870-020-02442-z/MediaObjects/12870_2020_2442_MOESM1_ESM.png) Total number of expressed genes for each sample. In this figure, MH: match-head, SM: square growth midpoint, 1DPA: a day post anthesis ovule, maternal parent, paternal parent and F1 are represented in different colour (three biological replicates).

[**Figure S5.**](https://static-content.springer.com/esm/art%3A10.1186%2Fs12870-020-02442-z/MediaObjects/12870_2020_2442_MOESM1_ESM.png)


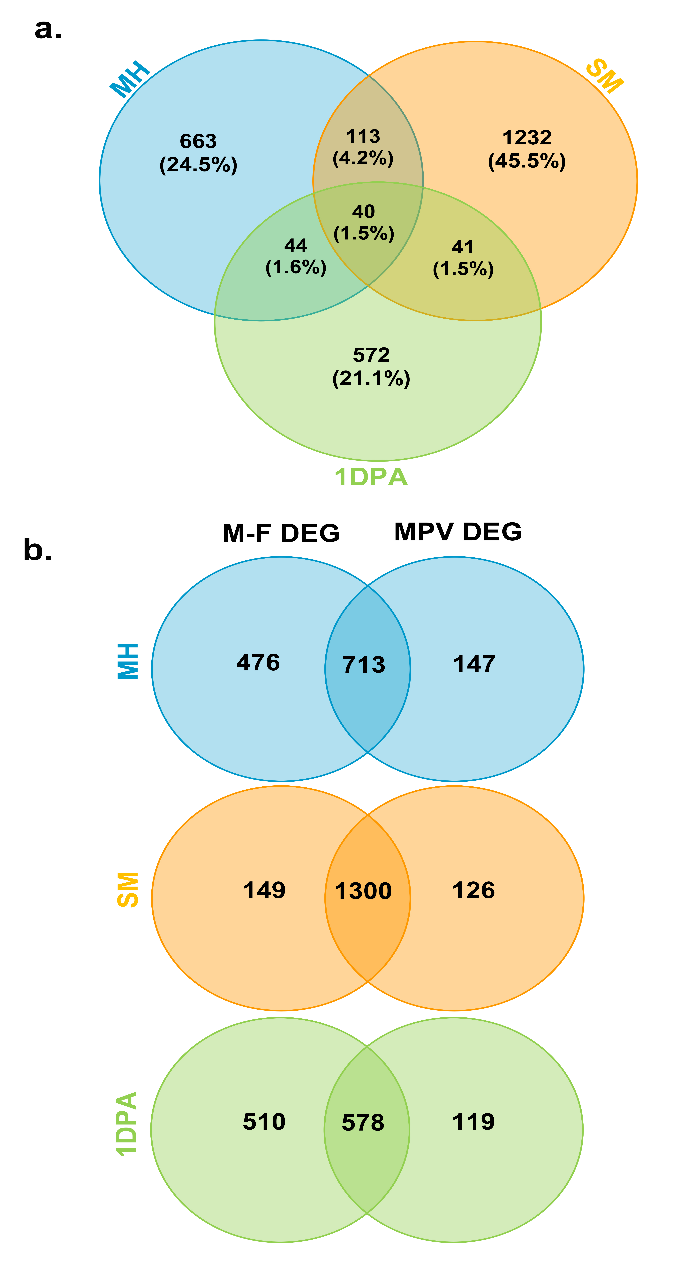


[**Figure S5.**](https://static-content.springer.com/esm/art%3A10.1186%2Fs12870-020-02442-z/MediaObjects/12870_2020_2442_MOESM1_ESM.png) (a) Breakdown of DEGs in the F1 hybrid showing the number of differentially expressed genes that were unique in one sample or overlapping in two or three samples used in this study. (b) Overlapping genes between MPV-DEGs and M-F DEGs in all three developmental stages.

**Figure S6.**


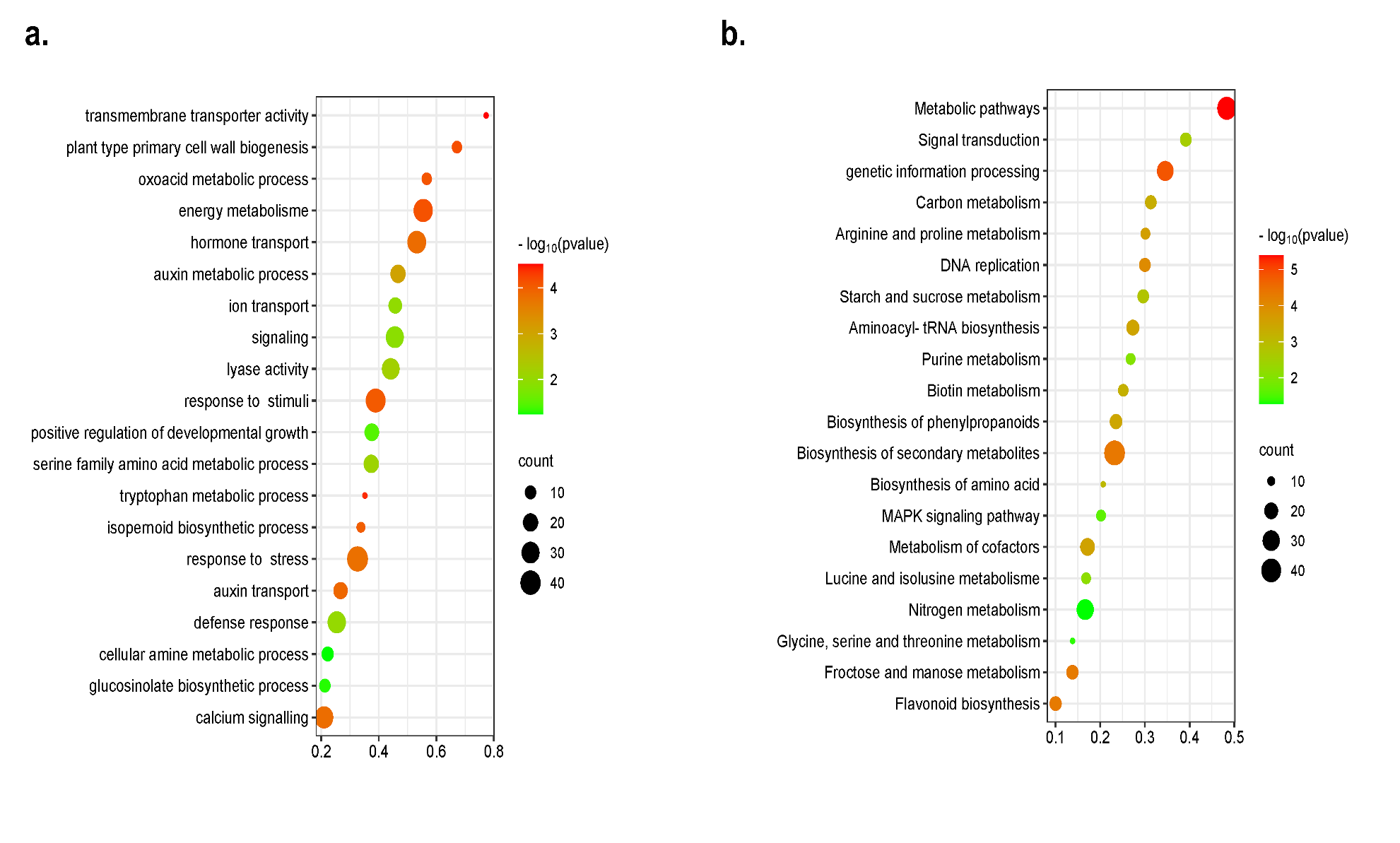


**Figure S6.** Prominent GO terms enriched in the genes with a trans effect. (a) Gene Ontology (GO) and (b) KEGG enrichment analyses for all trans effects in genes in all datasets ([www.kegg.jp/kegg/kegg1.html](http://www.kegg.jp/kegg/kegg1.html), For previous uses, the Kanehisa laboratory have happily provided permission).

**Figure S7.**


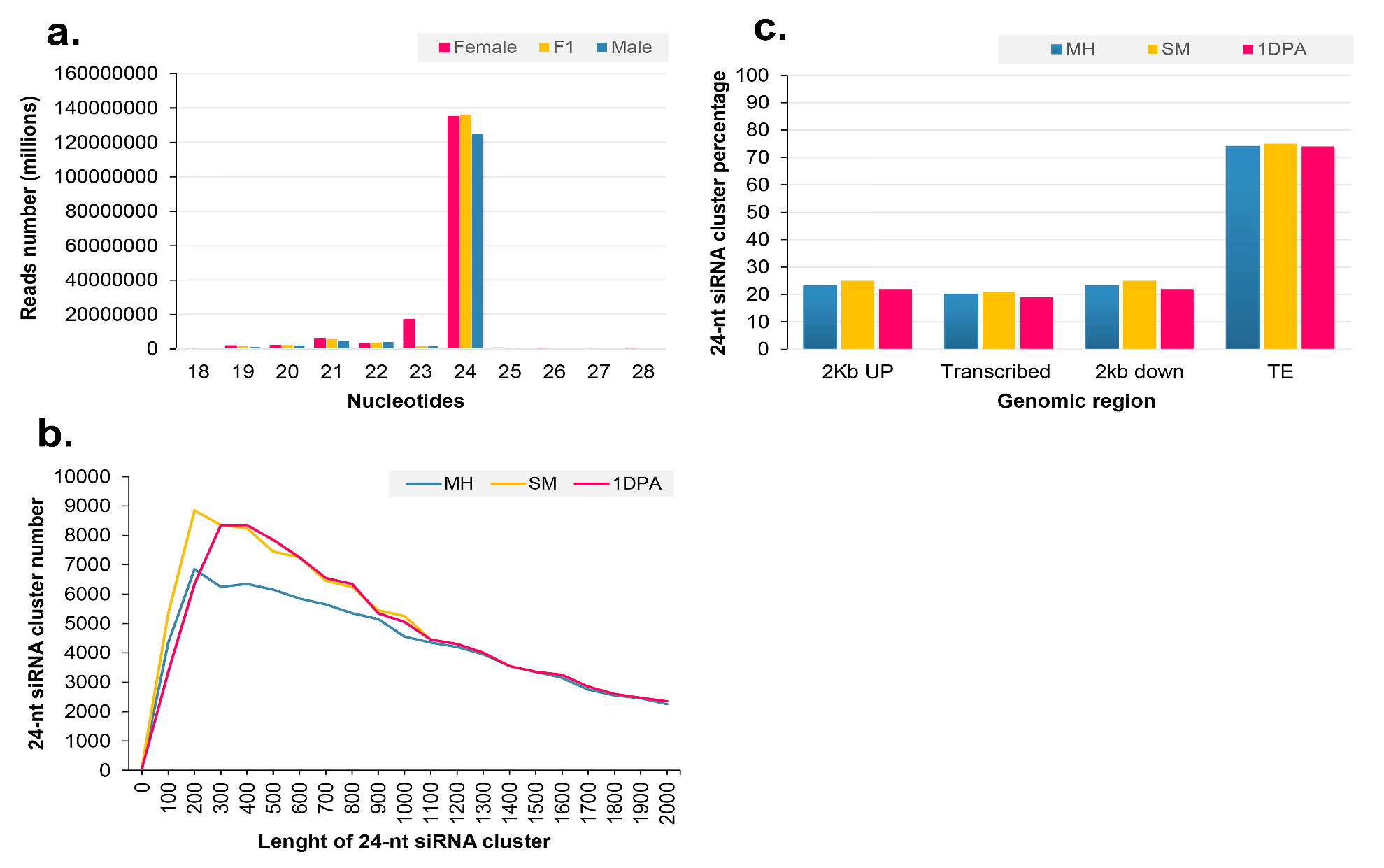


**Figure S7.** (a). Class distribution analysis of filtered reads in hybrid and parental lines showed that 21 and 24 nucleotide classes were the most abundant groups. (b) Distribution of the 24-nucleotide siRNA clusters in the 2 kb upstream, 2 kb downstream, transcribed region and transposable elements in the three samples. (c) Length distribution of the 24-nucleotide siRNA clusters in the three samples.

**Figure S8.**


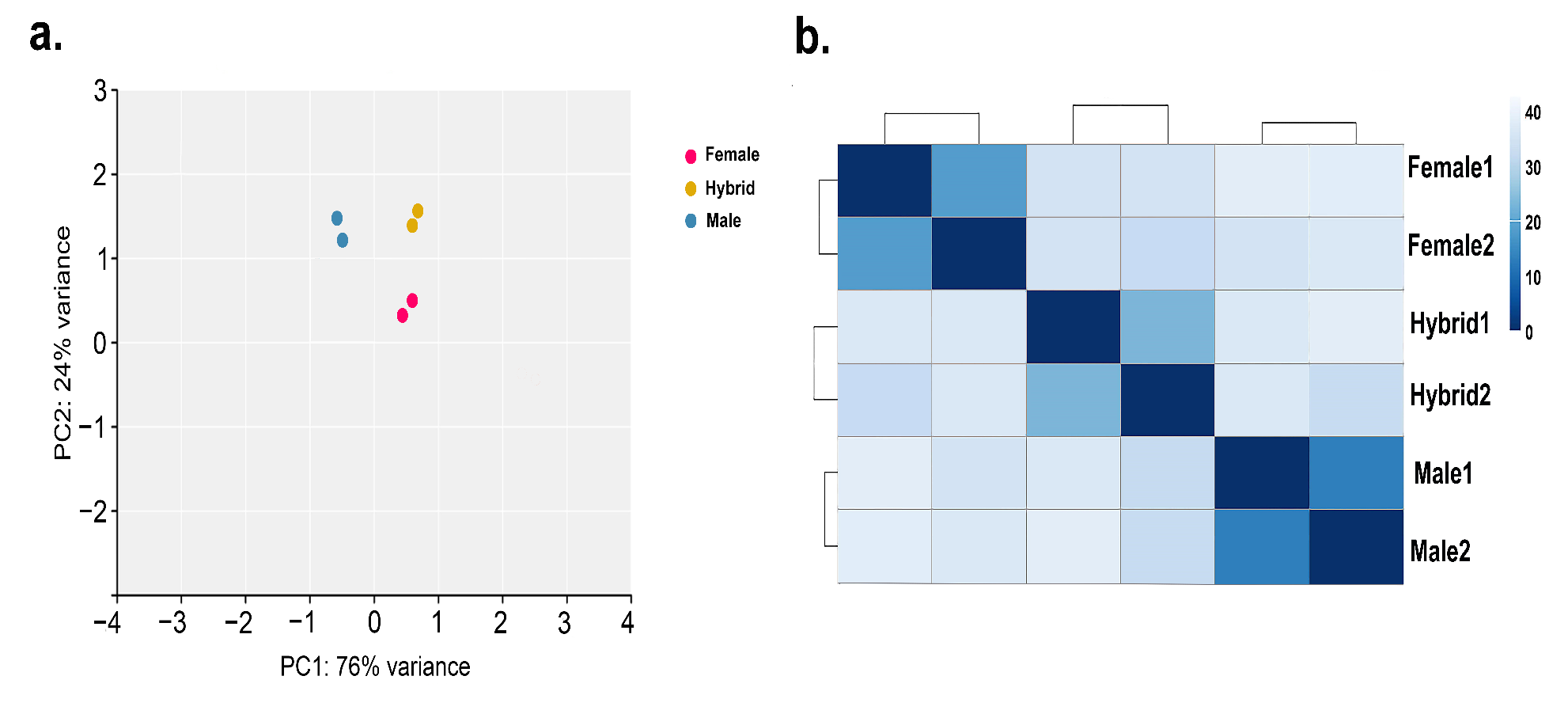


**Figure S8.** (a) Principal component analysis for all samples (two replicates). (b) Pearson correlation coefficients between different sample replicate used for whole-genome bisulfite sequencing (WGBS) analysis.

**Figure S9.**


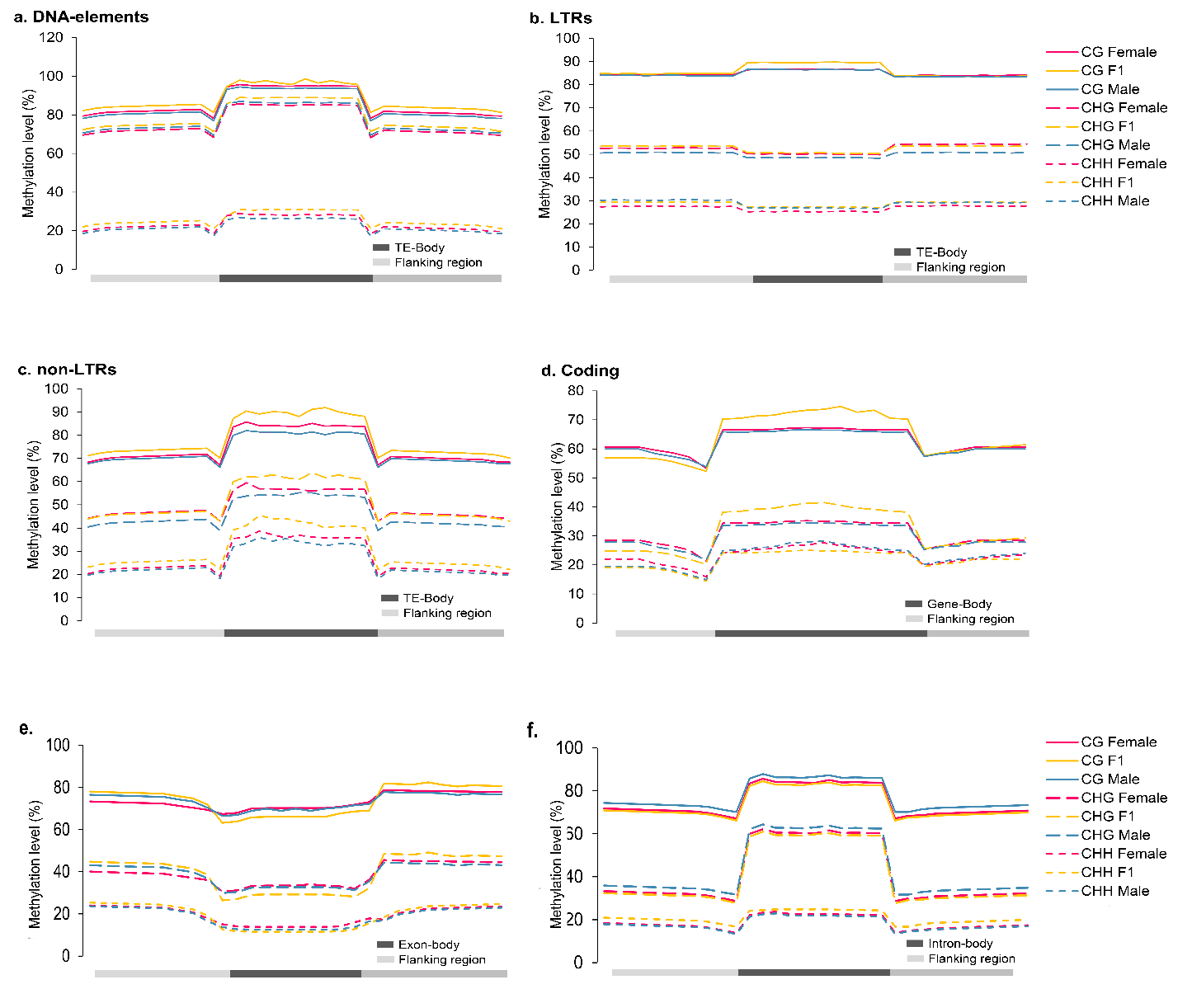


**Figure S9.** Average methylation distribution across different genomic regions. Metaplots representing methylation distribution at genomic elements and their flanking regions (a–d) transposable-elements (TEs) body including DNA-elements, LTRs, non-LTRs and gene-body, respectively, along with their 1.5 kb flanking regions. Methylation at gene/TE -body and flanking regions was analyzed across 20 and 30 bins, respectively. (e, f) exon-body and intron body methylation along with their 1.5 kb flanking regions.

**Figure S10.**


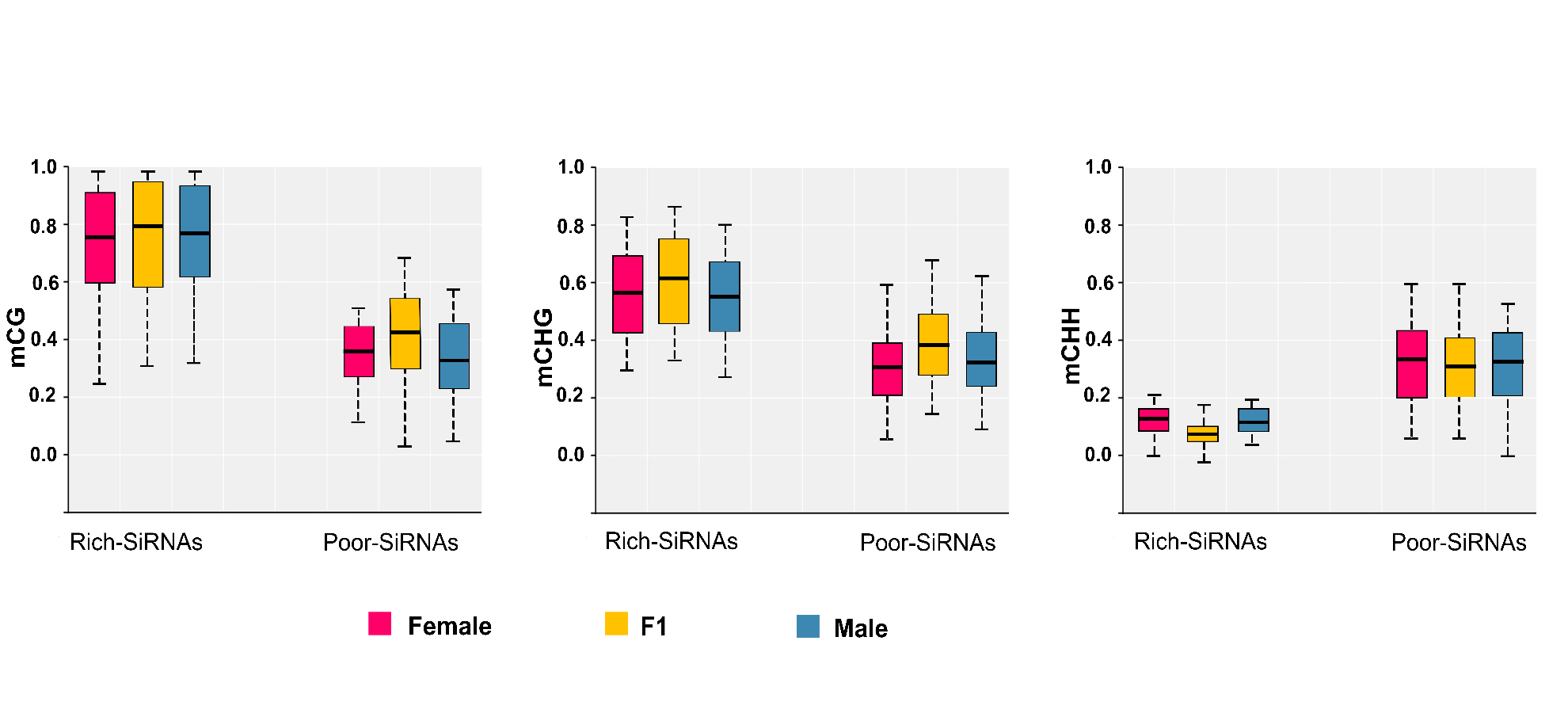


**Figure S10.** Boxplots showing the methylation levels of CG, CHG, and CHH on Rich-siRNA clusters and Poor-siRNA clusters in hybrid and its parent.

1. Kanehisa M: **Toward understanding the origin and evolution of cellular organisms**. *Protein Science* 2019, **28**(11):1947-1951.

2. Kanehisa M, Furumichi M, Sato Y, Kawashima M, Ishiguro-Watanabe M: **KEGG for taxonomy-based analysis of pathways and genomes**. *Nucleic acids research* 2023, **51**(D1):D587-D592.

3. Kanehisa M, Goto S: **KEGG: kyoto encyclopedia of genes and genomes**. *Nucleic acids research* 2000, **28**(1):27-30.
